# Seeking effective interventions to treat complex wounds: an overview of systematic reviews

**DOI:** 10.1186/s12916-015-0288-5

**Published:** 2015-04-22

**Authors:** Andrea C Tricco, Jesmin Antony, Afshin Vafaei, Paul A Khan, Alana Harrington, Elise Cogo, Charlotte Wilson, Laure Perrier, Wing Hui, Sharon E Straus

**Affiliations:** 1 grid.415502.7Li Ka Shing Knowledge Institute, St. Michael’s Hospital, 209 Victoria Street, East Building, Toronto, Ontario M5B 1W8 Canada; 2 grid.17063.33Epidemiology Division, Dalla Lana School of Public Health, University of Toronto, 155 College Street, Toronto, Ontario M5T 3M7 Canada; 3 grid.17063.33Department of Geriatric Medicine, University of Toronto, 27 Kings College Circle, Toronto, Ontario M5S 1A1 Canada

**Keywords:** Complex wound, Effectiveness, Systematic review, Treatment, Ulcer, Wounds

## Abstract

**Background:**

Numerous, often multi-faceted regimens are available for treating complex wounds, yet the evidence of these interventions is recondite across the literature. We aimed to identify effective interventions to treat complex wounds through an overview of systematic reviews.

**Methods:**

MEDLINE (OVID interface, 1946 until October 26, 2012), EMBASE (OVID interface, 1947 until October 26, 2012), and the Cochrane Database of Systematic Reviews (Issue 10 of 12, 2012) were searched on October 26, 2012. Systematic reviews that examined adults receiving care for their complex wounds were included. Two reviewers independently screened the literature, abstracted data, and assessed study quality using the Assessment of Multiple Systematic Reviews (AMSTAR) tool.

**Results:**

Overall, 99 systematic reviews were included after screening 6,200 titles and abstracts and 422 full-texts; 54 were systematic reviews with a meta-analysis (including data on over 54,000 patients) and 45 were systematic reviews without a meta-analysis. Overall, 44% of included reviews were rated as being of high quality (AMSTAR score ≥8). Based on data from systematic reviews including a meta-analysis with an AMSTAR score ≥8, promising interventions for complex wounds were identified. These included bandages or stockings (multi-layer, high compression) and wound cleansing for venous leg ulcers; four-layer bandages for mixed arterial/venous leg ulcers; biologics, ultrasound, and hydrogel dressings for diabetic leg/foot ulcers; hydrocolloid dressings, electrotherapy, air-fluidized beds, and alternate foam mattresses for pressure ulcers; and silver dressings and ultrasound for unspecified mixed complex wounds. For surgical wound infections, topical negative pressure and vacuum-assisted closure were promising interventions, but this was based on evidence from moderate to low quality systematic reviews.

**Conclusions:**

Numerous interventions can be utilized for patients with varying types of complex wounds, yet few treatments were consistently effective across all outcomes throughout the literature. Clinicians and patients can use our results to tailor effective treatment according to type of complex wound. Network meta-analysis will be of benefit to decision-makers, as it will permit multiple treatment comparisons and ranking of the effectiveness of all interventions.

Please see related article: http://dx.doi.org/10.1186/s12916-015-0326-3

**Electronic supplementary material:**

The online version of this article (doi:10.1186/s12916-015-0288-5) contains supplementary material, which is available to authorized users.

## Background

Chronic wounds are those that have not progressed through the ordered process of healing to yield a functional result [1]. Recently, the terminology for chronic wounds has changed. The preferred term to refer to a chronic wound is a “complex wound” [2]. One of the following characteristics is necessary for a wound to be classified as being complex: i) has not healed in 3 months, ii) infection is present, iii) compromised viability of superficial tissues, necrosis, or circulation impairment, and iv) association with systemic pathologies, impairing normal healing [2]. The main types of complex wounds include diabetic leg/foot ulcers, pressure ulcers [3], chronic venous ulcers, infected wounds [1,4,5], and those related to vasculitis and immunosuppressive therapy that have not healed using simple care [2].

Complex wounds are a significant burden on society. It has been estimated that complex wounds cost the healthcare system $10 billion annually in North America alone [6]. These estimates often fail to capture indirect costs, including patient/caregiver frustration, economic loss, and decreased quality of life.

Healthcare providers and patients have numerous regimens available for treating wounds [7], including dressings, wound cleansing agents, skin replacement therapy, biologic agents, stockings, nutritional supplementation, complementary and alternative medicine, bandages, and surgery, to name a few. Furthermore, wound care is often multi-faceted, and several interventions may be used concurrently. Some of these interventions have been examined in overviews of Cochrane reviews [8,9]. As the evidence of these interventions is recondite across the literature, we sought to elucidate optimal treatment strategies for complex wounds through an overview of all available systematic reviews, including Cochrane reviews and non-Cochrane reviews.

## Methods

### Protocol

A protocol for our overview of reviews was developed using the Cochrane Handbook for overviews of reviews [10]. The draft protocol was circulated for feedback from systematic review methodologists, policy-makers, and clinicians with expertise in wound care. It was revised as necessary and the final version is available from the corresponding author upon request.

### Eligibility criteria

We included systematic reviews that focused on interventions to treat complex wounds (including venous and arterial ulcers due to chronic illness, diabetic ulcers, pressure ulcers, and infected surgical wounds) amongst adults aged 18 years and older. We used the definition for a systematic review put forth by the Cochrane Collaboration, “*A systematic review attempts to collate all empirical evidence that fits pre-specified eligibility criteria in order to answer a specific research question. It uses explicit, systematic methods that are selected with a view to minimizing bias, thus providing more reliable findings from which conclusions can be drawn and decisions made*” [10].

A list of the 14 different intervention categories can be found in Additional file 1. All comparators, such as other wound care interventions, no treatment, placebo, and usual care were eligible for inclusion. To be included, a systematic review also had to report on our outcomes of interest as identified by decision-makers, including healing (e.g., number of ulcers healed, improvement of ulcers, and time to ulcer healing) or admission to hospital (including readmissions). Systematic reviews that were published or unpublished and conducted at any point in time were included. Due to resource limitations, only systematic reviews written in English were included. However, authors were contacted to obtain translations of reviews written in languages other than English.

### Literature search

Comprehensive literature searches were conducted from inception until October 2012 across MEDLINE, EMBASE, and the Cochrane Database of Systematic Reviews. The search terms included both medical subject headings (MeSH) and free text terms related to wound care interventions. Literature searches were conducted by an experienced librarian (LP) on October 26, 2012. Using validated search filters, the search strategies were limited to human participants, adults, and systematic reviews. The electronic database search was supplemented by searching for systematic review protocols in the PROSPERO database [11], contacting authors of conference proceeding abstracts for their unpublished data, and scanning the reference lists of the included systematic reviews.

The search strategy was peer reviewed by another expert librarian on our team (EC) using the Peer Review of Electronic Search Strategies checklist [12] and amended, as necessary. The final search strategy for the MEDLINE database is presented in Additional file 2. The literature search was limited to adults, reviews, and economic studies. The latter limitation was employed to identify cost-effectiveness analyses for a second paper that examines the cost-effectiveness of complex wounds [Tricco et al., unpublished paper submitted to *BMC Medicine*]. The MEDLINE search was modified for the other two databases, as necessary. Search strategies for the EMBASE and Cochrane Database of Systematic Reviews are available from the corresponding author, upon request.

### Screening

Prior to commencing the screening process, a calibration exercise was conducted to ensure reliability in correctly selecting articles for inclusion. This exercise entailed screening a random sample of 50 of the included titles and abstracts by all team members, independently. The eligibility criteria were modified, as necessary, to optimize clarity. Subsequently, reviewer pairs (ACT, JA, AH, AV, PAK, CW, EC, LP) independently screened the remainder of the search results for inclusion using a pre-defined relevance criteria form for all levels of screening (e.g., title and abstract, full-text review of potentially relevant articles). Discrepancies were resolved by discussion or the involvement of a third reviewer.

### Data items

Data abstraction forms were pilot-tested by all team members independently on a random sample of five articles. The data abstraction forms were revised after this exercise, as necessary. Subsequently, reviewer pairs (ACT, JA, AH, AV, PAK, CW, LP, WH) independently read each article and abstracted relevant data. Differences in abstraction were resolved by discussion or the involvement of a third reviewer. Data items included study characteristics (e.g., number of studies identified, type of study designs included, interventions and comparators examined), patient characteristics (e.g., clinical population, wound types, age category), and outcome results (e.g., healing, hospitalizations).

### Methodological quality appraisal

The methodological quality of the included systematic reviews were appraised using the Assessment of Multiple Systematic Reviews (AMSTAR) tool [13]. The reliability and validity of this tool has been established [14]. Items include the use of a protocol, study selection by two reviewers, comprehensive literature search, inclusion of unpublished material, list of included and excluded studies, reporting of study characteristics, quality appraisal, appropriate pooling of data methods, assessment of publication bias, and statement of conflicts of interest. Each included systematic review was appraised by two team members (ACT, JA, AH, AV, PAK, CW, EC, LP) and conflicts were resolved by discussion or the involvement of a third reviewer.

### Synthesis

Literature search results and the abstracted data were summarized descriptively. An in-depth comparison of included systematic reviews was compiled and depicted in tables and figures. Conclusion statements for systematic reviews without a meta-analysis were categorized by two reviewers (ACT, JA, AH, AV, PAK, CW, LP, WH), independently, using a pre-existing framework, as follows: positive (authors stated that there is evidence of effectiveness); neutral (no evidence of effectiveness or they reported no opinion); negative (authors advised against the use of the intervention or it was not recommended); or indeterminate (authors stated that there is insufficient evidence or that more research is required) [15]. Conflicts were resolved through discussion and a third reviewer (ACT) verified the categorizations to ensure accuracy.

## Results

### Literature search

**Figure 1:**
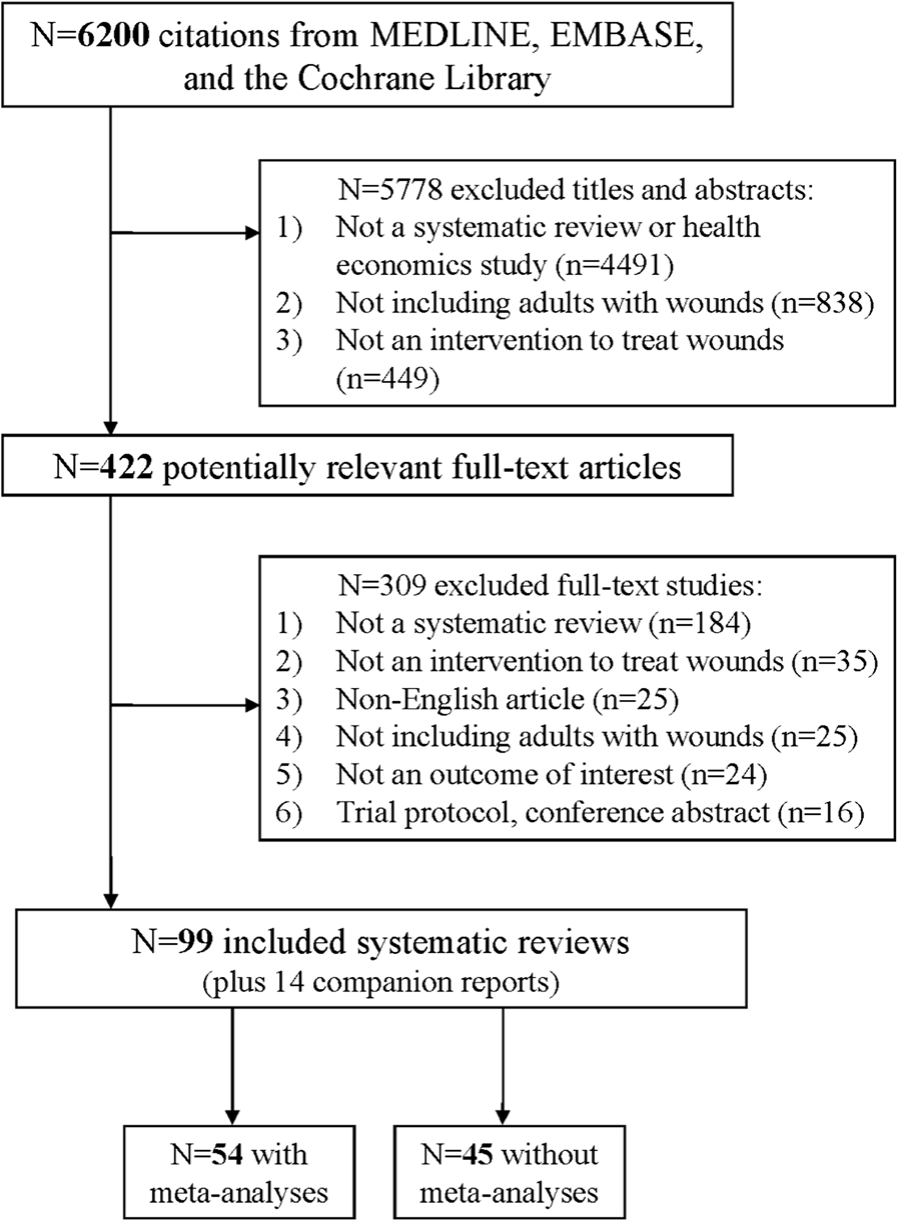
**Study flow.** Details the flow of information through the different phases of the review. The flow maps out the number of records identified, included and excluded, and the reasons for their exclusion.

The literature search resulted in 6,200 titles and abstracts, of which 5,778 were excluded for not fulfilling the eligibility criteria (Figure 1). Of the 422 full-texts retrieved and screened in duplicate, 309 articles were excluded. Ninety-nine systematic reviews of wound care interventions were included in this overview of systematic reviews; 54 were systematic reviews with meta-analysis results [16-67] and 45 were systematic reviews without a meta-analysis [68-112]. In addition, 14 companion reports were included [21,113-125], the majority of which were Cochrane updates.

### Systematic review characteristics

The reviews were conducted between 1997 and 2012, with 28% taking place after 2011 (Table 1; Additional file 3). The first authors of the systematic reviews were predominantly based in Europe (66%), North America (19%), and Australia or New Zealand (7%). The number of studies included in each review ranged from 0 to 130, with 80% including between 2 and 30 studies. Ninety-three systematic reviews included randomized clinical trials. Five systematic reviews were unpublished [43,58,83,104,106].

**Table 1:** **Summary characteristics of included systematic reviews**

**Characteristic**	**Number of systematic reviews (n = 99)**	**Percentage of systematic reviews**
**Year**		
1997–1999	6	6.1
2000–2002	9	9.1
2003–2005	15	15.2
2006–2008	21	21.2
2009–2011	36	36.4
2012	12	12.1
**Country of conduct**		
Europe (38 of these are from the United Kingdom)	65	65.7
North America	19	19.2
Australasia (Australia, New Zealand)	7	7.1
Asia (Malaysia, China, Taiwan, India)	6	6.1
South America	2	2.0
**Number of studies included**		
0–1	3	3.0
2–10	53	53.5
11–20	16	16.2
21–30	10	10.1
31–40	6	6.1
41–100	8	8.1
>100	3	3.0
**Study designs included** ^*^		
Randomized clinical trials	93	70.5
Observational studies		
Non-randomized clinical trials	20	15.2
Controlled before-after studies, interrupted	17	12.9
Time series	2	1.5
**Patient population**		
Not specifically reported	65	65.7
Diabetes	21	21.2
Chronic venous disease	4	4.0
Complex lower limb wounds	4	4.0
Inpatients/institutionalized	3	3.0
Ambulatory patients	1	1.0
Elderly	1	1.0
**Type of wound** ^*^		
Venous leg ulcers	36	31.3
Diabetic foot/leg ulcers	26	22.6
Pressure ulcers	20	17.4
Mixed arterial/venous leg ulcers	16	13.9
Mixed complex wound unspecified	10	8.7
Infected surgical wounds	7	7.0
**Interventions examined** ^*^		
Adjuvant	33	20.3
Dressings	26	16.0
Biologics	16	9.8
Other topical	14	8.6
Other oral	11	6.8
Stockings	10	6.1
Support surfaces	10	6.1
Wound cleansing	10	6.1
Skin replacement	9	5.5
Bandages	7	4.3
Surgery	7	4.3
Nutritional supplementation	4	2.5
Wound care program	4	2.5
Complementary and alternative medicine	2	1.2
**Comparators examined** ^*^		
Usual care	63	45.7
Dressings	34	24.6
Bandages	8	5.8
Not reported	8	5.8
Support surfaces	7	5.1
Other topical	5	3.6
Wound cleansing	5	3.6
Stockings	4	2.9
Other oral	2	1.5
All other treatments	1	0.7
Skin replacement	1	0.7
**Number of treatment comparisons per outcome**		
*Systematic reviews with a meta-analysis*	n = 143 comparisons	%
Wound area/size reduction	10	7.0
Time to healing or rate of healing	10	7.0
Ulcer healing	20	14.0
Proportion of patients with healed wounds	97	67.8
No healing improvement	5	3.5
Length of hospitalization	1	0.7
*Systematic reviews without a meta-analysis*	n = 184 comparisons	%
Wound area/size reduction	18	9.8
Time to healing or rate of healing	53	28.8
Ulcer healing	92	50.0
Proportion of patients with healed wounds	21	11.4

^*^Numbers do not add up to 99, as the systematic reviews contributed data to more than one category.

### Study and patient characteristics

Thirty-four systematic reviews provided information about the patient population under study (Table 1; Additional file 3) and 21% included patients with diabetes. Six categories of complex wounds were examined: venous leg ulcers (31%), diabetic foot/leg ulcers (23%), pressure ulcers (17%), mixed arterial/venous leg wounds (14%), unspecified mixed complex wounds (9%), and infected surgical wounds (6%). The five most common interventions were adjuvant therapies (20%), dressings (16%), biologic agents (10%), other topical agents (9%), and other oral agents (7%). The five most common comparators were usual care (46%), dressings (25%), bandages (6%), support surfaces (5%), and other topical agents (4%). The duration of treatment ranged from 2 days to 160 months and the duration of follow-up ranged from 2 days to 195 months across the included studies in the systematic reviews.

A total of 327 treatment comparisons were included in the 99 systematic reviews. As such, only statistically significant results from systematic reviews with a meta-analysis are reported in our outcome results section below in the text. Specific results for all treatment comparisons can be found in Table 2. To facilitate the summary and comparison of a large number of reviews, we have presented the review results using positive, negative, or neutral conclusions (Tables 2,3,4,5,6,7); however, we have also included the statistical effect sizes from each of the included meta-analyses in Additional file 4.

**Table 2:** **Summary of evidence for venous ulcer management**

**Outcome**	**Intervention**	**Systematic reviews with MA**		**Systematic reviews without MA**	
		High-quality*	**Low/moderate quality**	High-quality**	**Low/moderate quality**
**Wound size reduction** (MAs: 2; non-MAs: NA) [36,56]	Cadexomer iodine (topical)	+	NA	NA	NA
	Micronized purified flavonoid fraction (MPFF) (oral)	NA	+	NA	NA
**Time to healing or rate of healing** (MAs: 5 [34,39,44,45,56]; non-MAs: 1 [77])	Four-layer bandage	+	NA	NA	NA
	MPFF (oral)	NA	+	NA	NA
	Stockings	NA	+	NA	+/−
	Silver-impregnated dressings	NA	–	NA	NA
	Applied freeze-dried keratinocyte lysate (topical)	NA	NA	NA	–
	Collagenase (topical)	NA	NA	NA	+/−
	Compression stockings	NA	NA	NA	+
	Flavonoids + compression	NA	NA	NA	+/−
	Intermittent pneumatic compression + compression	NA	NA	NA	+/−
	Larval therapy	NA	NA	NA	+/−
	Laser therapy	NA	NA	NA	+/−
	Leg ulcer clinics, wound care program	NA	NA	NA	+/−
	Multi-layer elastomeric high-compression	NA	NA	NA	–
	Platelet-derived growth factor (oral)	NA	NA	NA	+/−
	Rutosides (oral)	NA	NA	NA	+/−
	Semi-occlusive dressings: foam, film, hyaluronic acid-derived dressings, collagen, cellulose, or alginate	NA	NA	NA	–
	Stockings: multi-layer elastic system, multi-layer elastomeric (or non-elastomeric) high-compression regimens	NA	NA	NA	–
	Sulodexide (oral) + compression	NA	NA	NA	+
	Thromboxane α2 antagonists (oral)	NA	NA	NA	+/−
	Topical negative pressure (vacuum-assisted closure)	NA	NA	NA	+
	Ultrasound	NA	NA	NA	–
**Ulcer healing** (MAs: 8 [29,30,39,51,62]; non-MAs: 5 [72,73,77,89,103,119])	Elastic high compression bandages	+	NA	NA	+
	Cryopreserved allografts (Skin grafting)	NA	–	NA	+/−
	Cultured keratinocytes/epidermal grafts (Skin grafting)	NA	–	NA	+/−
	Fresh allografts (Skin grafting)	NA	–	NA	+/−
	Granulocyte-macrophage colony stimulating factor (topical)	NA	+	NA	NA
	Pentoxifylline (oral)	+	NA	NA	NA
	Stockings	NA	+	NA	
	Tissue engineered skin	NA	+	NA	NA
	Electromagnetic therapy	NA	NA	+	NA
	Hydrocolloid (occlusive) dressings + compression	NA	NA	NA	–
	Intermittent pneumatic compression (Flowtron, sequential gradient Jobst extremity pump)	NA	NA	NA	+/−
	Maggot debridement therapy	NA	NA	NA	+
	Mesoglycan (topical)	NA	NA	NA	+/−
	Superficial venous surgery	NA	NA	+	NA
**Proportion of patients with healed wounds** (*MAs: 39* [21,23,25,30,31,36,40,45,46,48,52,53,55,58,62,64-67]*;* *non-MAs: 2* [77,102])	Four-layer bandage	–	NA		
	Any laser (unspecified low level laser, ultraviolet therapy, non-coherent unpolarized red light)	NA	–	NA	NA
	Artificial skin graft and standard wound care	NA	–	NA	NA
	Autologous platelet-rich plasma (topical)	NA	–	NA	–
	Cadexomer iodine (topical) plus compression therapy	+	NA	NA	NA
	Systemic ciprofloxacin (oral)	NA	–	NA	NA
	Skin replacement therapy (Dermagraft)	NA	+	NA	+
	Elastic high compression bandages	+	NA	NA	NA
	Foam dressing	–	NA	NA	NA
	Granulocyte-macrophage colony stimulating factor (perilesional injection)	NA	+	NA	+
	High frequency ultrasound	–	NA	NA	NA
	Honey (topical)	–	NA	NA	NA
	Hydrocolloid dressings	–	NA	NA	NA
	Hydrogel dressing	–	NA	NA	NA
	Intermittent pneumatic compression stockings	+/−	–	NA	NA
	Low frequency ultrasound	–	NA	NA	NA
	Multi-layer high compression bandages	+/−	NA	NA	+
	Pentoxifylline (oral) with and without compression	+	NA	NA	+
	Stockings	+	NA	NA	+/−
	Two-component (outer elastic) bandages	+/−	NA	NA	NA
	Ultrasound	NA	–	NA	NA
	Unna’s boot	NA	–	NA	NA
	Zinc (oral)	–	NA	NA	NA
	Antimicrobial (topical)	NA	NA	NA	–
	Calcitonin gene-related peptide (topical)	NA	NA	NA	–
	Prostaglandin EI (IV)	NA	NA	NA	+
	Subfascial endoscopic perforator surgery	NA	NA	NA	+
	Superficial vein surgery	NA	NA	NA	+/−
	Systemic mesoglycan (IM, oral) + compression	NA	NA	NA	+

*At least one systematic review with meta-analysis and AMSTAR score ≥8.

**At least one systematic review without meta-analysis and AMSTAR score ≥8.

+ Effective (statistically significant difference between interventions and comparators); – No difference (no statistically significant difference between interventions and comparators); +/− Unknown (conflicting evidence between meta-analysis or indeterminate results); NA, No studies available; MA, Meta-analysis.

**Table 3:** **Summary of evidence for mixed arterial/venous ulcer management**

**Outcome**	**Intervention**	**Systematic reviews with MA**		**Systematic reviews without MA**	
		High-quality*	**Low/moderate quality**	High-quality**	**Low/moderate quality**
**Wound area/size reduction** (MAs: 3) [34,65]	Silver treatments (topical) and silver-impregnated dressings	NA	+/−	NA	NA
	Ultrasound	NA	+	NA	NA
**Ulcer healing** (MAs: NA; non-MAs: 6) [80,82-84,101,110]	Antimicrobial (topical and oral)	NA	NA	NA	–
	Electromagnetic therapy	NA	NA	NA	+
	Honey (topical)	NA	NA	NA	+/−
	Ketanserin ointment, 2% (topical)	NA	NA	NA	+/−
	Standardized wound treatment protocol	NA	NA	NA	+
	Silver releasing dressing	NA	NA	–	NA
**Time to healing or rate of healing** (MAs: 5) [45,49,55,56]	Topical negative pressure	NA	+	NA	NA
	Four-layer bandage	+	NA	NA	NA
	Micronized purified flavonoid fraction (oral)	NA	+	NA	NA
	Micronized purified flavonoid fraction (oral)	NA	+	NA	NA
	Polyurethane (dressing)	–	NA	NA	NA
	Alginate (beads, paste + dressing, alginate dressing)	–	NA	NA	NA
**Proportion of patients with healed wounds** (MAs: 3) [49,50]	Silver dressings (topical or impregnated)	NA	–	NA	NA
	Topical negative pressure	NA	+	NA	NA

*At least one systematic review with meta-analysis and AMSTAR score ≥8.

**At least one systematic review without meta-analysis and AMSTAR score ≥8.

+ Effective (statistically significant difference between interventions and comparators); – No difference (no statistically significant difference between interventions and comparators); +/− Unknown (conflicting evidence between meta-analysis or indeterminate results); NA, No studies available; MA, Meta-analysis.

**Table 4:** **Summary of evidence for diabetic ulcer management**

**Outcome**	**Intervention**	**Systematic reviews with MA**		**Systematic reviews without MA**	
		High-quality*	**Low/moderate quality**	High-quality**	**Low/moderate quality**
**Wound area/size reduction** (MAs: NA; non-MAs: 2) [79,88]	Hyperbaric oxygen therapy (systemic + usual care)	NA	NA	NA	–
	Stem cell therapy	NA	NA	NA	+
**Time to healing or rate of healing** (MAs: NA; non-MAs: 3)	Human skin equivalent	NA	NA	NA	+
	Human cultured dermis	NA	NA	NA	–
	Laser therapy and complex intervention	NA	NA	NA	+/−
[79,88,95]	Platelet derived growth factors (topical)	NA	NA	NA	+
	Pressure off-loading, felted foam	NA	NA	NA	–
	Pressure off-loading, total contact or non-removable cast	NA	NA	NA	+
	Stem cell therapy	NA	NA	NA	–
	Skin grafts	NA	NA	NA	–
	Topical negative pressure	NA	NA	–	NA
**Ulcer healing** (MAs: 4 [24,35,57]; non-MAs: 10 [69,70,74,79,81,86,88,93,112,122])	Chinese herbal medicine + standard therapy	NA	+	NA	NA
	Granulocyte colony-stimulating factor (SC, IV) + antibiotics (oral, IV)	+/−	–	NA	+/−
	Alginate, foam, hydrogel, hydrocolloid dressings	NA	NA	NA	+/−
	Alginate, hydrogel, hydrocellulose, semi-permeable membrane dressings	NA	NA	NA	–
	Amoxicillin + clavulanic acid (oral), ofloxacin, imipenem/cilastatin, ampicillin/sulbactam (IV)	NA	NA	NA	+/−
	Antibiotics, choice based on bone biopsy (IV, oral)	NA	NA	NA	+/−
	Ayurvedic preparations (oral + topical)	NA	NA	NA	+/−
	Clindamycin, fluoroquinolone, rifampicin, amoxicillin/clavulanic acid (oral, topical) +/− surgical intervention	NA	NA	NA	+/−
	Compression	NA	NA	NA	+
	Cultured human dermis	NA	NA	NA	+/−
	Dressings + debridement (hydrogel)	NA	NA	NA	+
	Early surgical intervention + antibiotics	NA	NA	NA	+/−
	Electrical stimulation	NA	NA	NA	–
	Endovascular or open bypass revascularization surgery of an ulcerated foot	NA	NA	NA	+/−
	Foot care clinic interventions	NA	NA	NA	+/−
	Growth factors (topical)	NA	NA	NA	–
	Hydrogel, cadexomer iodine ointment, dressings, larval therapy, sugar (topical) systemic antibiotics	NA	NA	NA	+/−
	Hyperbaric oxygen therapy	NA	NA	NA	+/−
	Hyperbaric oxygen therapy (systemic + usual care)	NA	NA	NA	–
	Imipenem/cilastatin, cefazolin, Ampicillin/sulbactam, Linezolid, Piperacillin/tazobactam. Amoxycillin + clavulanic acid, clindamycin hydrochloride (oral), pexiganan cream	NA	NA	–	NA
	Ketanserin (oral, topical)	NA	NA	NA	+/−
	Larval therapy	NA	NA	NA	+
	Lyophilized collagen, platelets and derived products (topical)	NA	NA	NA	+
	Magnet and normothermic therapy	NA	NA	NA	–
	Patient education	NA	NA	NA	+/−
	Percutaneous flexor tenotomy	NA	NA	NA	+/−
	Procaine + polyvinylpyrrolidone (IM)	NA	NA	NA	–
	Resection of the complex wound	NA	NA	NA	–
	Sharp debridement	NA	NA	NA	+/−
	Skin grafts	NA	NA	NA	+
	Stem cell therapy	NA	NA	NA	+
	Superoxidized water and soap, povidone iodine (topical)	NA	NA	NA	+/−
	Therapeutic footwear	NA	NA	NA	+/−
	Thrombin-induced human platelet growth factor, recombinant platelet derived growth factor, recombinant basic fibroblast growth factor,arginine-glycine-aspartic acid peptide matrix (topical)	NA	NA	NA	+/−
	Topical negative pressure	NA	NA	NA	+
	Negative pressure therapy	NA	NA	NA	+
	Total contact casting	NA	NA	NA	+/−
	Ultrasound	NA	NA	NA	–
	Zinc oxide tape	NA	NA	NA	+
**No healing improvement/non-healed wounds** (MAs: 5)	Chinese herbal medicine	NA	+	NA	NA
[22,33,35]	Hyaluronic acid-based scaffold and keratinocytes	–	NA	NA	NA
	Hyaluronic acid-based derivative	+	NA	NA	NA
	Low frequency low intensity noncontact ultrasound	+	NA	NA	NA
**Proportion of patients with healed wounds** (MAs: 18) [17-20,22,24,27,28,35,38,46,47,57-59]	Alginate dressing	–	NA	NA	NA
	Artificial skin graft and standard care	NA	+	NA	NA
	Chinese herbal medicine plus standard treatment	NA	+	NA	NA
	Skin graft (Dermagraft)	NA	–	NA	NA
	Fibrous-hydrocolloid (hydrofibre) dressing	–	NA	NA	NA
	Foam dressing	–	NA	NA	NA
	Granulocyte colony-stimulating factor (SC, IV) + antibiotics (oral, IV)	+	+	NA	NA
	Hyaluronic acid-based scaffold and keratinocytes	–	NA	NA	NA
	Hydrogel dressing	+	+	NA	NA
	Hyperbaric oxygen therapy	–		NA	NA
	Platelet-rich plasma	NA	+	NA	NA
	Skin replacement therapy (keratinocyte allograft, meshed skin autograft, split thickness autograft)	NA	+	NA	NA

*At least one systematic review with meta-analysis and AMSTAR score ≥8.

**At least one systematic review without meta-analysis and AMSTAR score ≥8.

+ Effective (statistically significant difference between interventions and comparators); – No difference (no statistically significant difference between interventions and comparators); +/− Unknown (conflicting evidence between meta-analysis or indeterminate results); NA, No studies available; MA, Meta-analysis.

**Table 5:** **Summary of evidence for pressure ulcer management**

**Outcome**	**Intervention**	**Systematic reviews with MA**		**Systematic reviews without MA**	
		High-quality*	**Low/moderate quality**	High-quality**	**Low/moderate quality**
**Wound area/size reduction** (MAs: NA; non-MAs: 3) [78,87,94]	Air-fluidized support	NA	NA	NA	+
	Alternating pressure mattress, low-air-loss mattress, air-fluidized mattress	NA	NA	NA	–
	Collagenase	NA	NA	NA	+/−
	Collagenase, hydrogel dressings	NA	NA	NA	–
	Electric current, electromagnetic therapy	NA	NA	NA	–
	Foam, calcium alginate, radiant heat dressing, dextranomer powder dressings	NA	NA	NA	–
	Hydrocolloid dressings	NA	NA	NA	+
	Hydrocolloid, hydrogel wafer, hydrogel, occlusive polyurethane, transparent moisture-permeable dressings	NA	NA	NA	–
	Hydrogel, cadexomer iodine, semelil gel, radiant heat, zinc salt spray, aluminum hydroxide, vitamin A ointment, streptokinase-streptodornase, dialysate, topical insulin, moist saline gauze and whirlpool, semelil dressings	NA	NA	NA	–
	Low level laser therapy, laser and standard care	NA	NA	NA	–
	Polarized light, monochromatic light and cadexomer iodine or hydrocolloid	NA	NA	NA	–
	Ultrasound	NA	NA	NA	–
	Vacuum therapy	NA	NA	NA	–
	Vitamin C and ultrasound, consistent wound care and controlled nutritional support, vitamin C, zinc sulfate	NA	NA	NA	–
**Time to healing or rate of healing** (MAs: NA; non-MAs: 3) [68,78,94]	Ascorbic acid, high-protein diet, concentrated, fortified, collagen protein hydrolysate supplement, disease-specific nutrition treatment	NA	NA	NA	+/−
	Amorphous hydrogel dressing derived from Aloe vera wound dressings	NA	NA	+/−	NA
	Electromagnetic therapy, low-intensity direct current, negative-polarity and positive-polarity electrotherapy, and alternating-polarity electrotherapy	NA	NA	NA	+/−
	Hydrocolloid dressings	NA	NA	NA	+/−
	Low air-loss beds	NA	NA	NA	+/−
	Low level laser therapy	NA	NA	NA	+/−
	Low-tech constant low-pressure supports	NA	NA	NA	+/−
	Phenytoin ointment (topical)	NA	NA	NA	+/−
	Seat cushions	NA	NA	NA	+/−
	Topical negative pressure	NA	NA	NA	+/−
	Triple antibiotic ointment, active cream dressings	NA	NA	NA	–
	Ultrasound	NA	NA	NA	–
**Ulcer healing** (MAs: 8 [16,43,60,62,64]; non-MAs: 11 [75,76,78,85,90,92,94,97,99,104,106])	Air-fluidized bed/supports	+	+	+/−	NA
	Air-fluidized beds, air suspension beds, foam replacement mattress	NA	NA	–	NA
	Alternating pressure surfaces	NA	NA		+/−
	Alternating pressure surfaces (alternating pressure mattress + pressure relief cushion)	NA	NA	+/−	NA
	Ascorbic acid, zinc sulfate	NA	NA	+/−	NA
	Collagen protein, standard hospital diet and high protein, standard hospital diet and high protein and zinc and arginine and vitamin C	NA	NA	NA	–
	Collagenase (topical)	NA	NA	NA	+
	Fibroblast-derived dermal replacement	NA	NA	NA	+
	Hydrocolloid, polyurethane, dextranomer, hydrogel, polyhydroxyethyl methacrylate, amino acid copolymer dressings	NA	NA	NA	–
	Low air-loss mattress, alternating pressure mattress, air-fluidized mattress	NA	NA	NA	–
	Phenytoin solution, antibiotics dressings	NA	NA	NA	–
	Protease-modulating matrix, recombinant platelet-derived growth factor BB, nerve growth factor, transforming growth factor beta, granulocyte-macrophage/colony stimulating factor, basic fibroblast growth factor (topical)	NA	NA	NA	+
	Saline spray containing Aloe vera, silver chloride and decyl glucoside, saline, whirlpool	NA	NA	NA	–
	Topical negative pressure	NA	NA	NA	–
	Topical negative pressure (vacuum assisted wound closure)	NA	NA	NA	–
	Alternative foam mattress	+	NA	NA	NA
	Electrotherapy	+	NA	NA	NA
	High protein, oral nutritional support, enteral tube feeding	–	NA	NA	NA
	Hydrocolloid dressings	+	NA	NA	NA
	Polyurethane dressings	–	NA	NA	NA
**Proportion of patients with healed wounds** (MAs: 25 [43,54,62]; non-MAs: 2 [78,94])	Collagenase debridement (topical)	NA	–	NA	NA
	Dextranomer (beads + dry dressing)	NA	–	NA	NA
	Electrical stimulation	NA	–	NA	NA
	Electrotherapy	NA	–	NA	–
	Growth Factors (topical)	NA	–	NA	
	Hydrocolloid dressings	NA	+/−	NA	–
	Hydrogel (gel)	NA	+	NA	–
	Hydropolymer dressing	NA	–	NA	NA
	Low air-loss beds	–	–	NA	NA
	Low level laser therapy	NA	–	NA	NA
	Alternating pressure mattress	NA	+/−	NA	NA
	Non-contact normothermic dressing	NA	–	NA	NA
	Polyurethane dressings	NA	–	NA	–
	Ultrasound	–	–	NA	+/−
	Zinc supplement (oral)	NA	–	NA	
	Collagenase, dressings	NA	NA	NA	–
	Laser therapy + moist saline gauze	NA	NA	NA	–
	Monochromatic phototherapy, UV light	NA	NA	NA	–
	Oxyquinoline, radiant heat, soft silicone, hydrogel or foam, active ointment with live yeast derivative, topical insulin (dressings)	NA	NA	NA	–
	Resin salve absorbent dressings	NA	NA	NA	–
	Specialized foam mattress, alternating pressure mattress	NA	NA	NA	–

*At least one systematic review with meta-analysis and AMSTAR score ≥8.

**At least one systematic review without meta-analysis and AMSTAR score ≥8.

+ Effective (statistically significant difference between interventions and comparators); – No difference (no statistically significant difference between interventions and comparators); +/− Unknown (conflicting evidence between meta-analysis or indeterminate results); NA, No studies available; MA, Meta-analysis.

**Table 6:** **Summary of evidence for mixed complex wounds management**

**Outcome**	**Intervention**	**Systematic reviews with MA**		**Systematic reviews without MA**	
		High-quality*	**Low/moderate quality**	High-quality**	**Low/moderate quality**
**Wound area/size reduction** (MAs:5) [21,32,41,42]	Autologous platelet-rich plasma/platelet-rich plasma (topical)	–	–	NA	NA
	Silver releasing dressings	+	NA	NA	NA
	Topical negative pressure	NA	+	NA	NA
**Ulcer healing** (MA: 1 [63]; non-MAs: 5 [91,100,105,107,111])	Laser therapy	NA	–	NA	NA
	Adhesive zinc oxide tape	NA	NA	+	NA
	Dextranomer polysaccharide beads or paste, cadexomer iodine polysaccharide beads or paste	NA	NA	_	NA
	Enzymatic agents (topical)	NA	NA	–	NA
	Hydrogel dressings	NA	NA	–	NA
	Hyperbaric oxygen therapy	NA	NA	NA	+
	No-sting barrier film bandages	NA	NA	NA	+
	Silver releasing dressing, non-releasing silver-activated charcoal dressing, hydrocolloid silver Vaseline-impregnated dressing, silver coated dressing, hydrocolloid silver-releasing dressing, silver-releasing foam dressing	NA	NA	+	NA
	Topical negative pressure (open-cell foam dressing with continuous suction)	NA	NA	+	NA
**Proportion of patients with healed wounds** (MAs: 10) [21,55,58,61,62,125]	Skin replacement (skin substitute) and standard care	NA	+	NA	NA
	Skin replacement (dermal substitute) and standard care	NA	+	NA	NA
	Artificial skin grafts and standard care	NA	+	NA	NA
	Autologous platelet-rich plasma/platelet-rich plasma (topical)	NA	–	NA	NA
	Hydrocolloid dressings	–	+	NA	NA
	Laser therapy	–	NA	NA	NA
	Ultrasound	+/−	NA	NA	NA

*At least one systematic review with meta-analysis and AMSTAR score ≥8.

**At least one systematic review without meta-analysis and AMSTAR score ≥8.

+ Effective (statistically significant difference between interventions and comparators); – No difference (no statistically significant difference between interventions and comparators); +/− Unknown (conflicting evidence between meta-analysis or indeterminate results); NA, No studies available); MA, Meta-analysis.

**Table 7:** **Summary of evidence for infected surgical wounds management**

**Outcome**	**Intervention**	**Systematic reviews with MA**		**Systematic reviews without MA**	
		High-quality*	**Low/moderate quality**	High-quality**	**Low/moderate quality**
**Proportion of patients with healed wounds** (MAs: 1) [37]	Topical negative pressure/vacuum-assisted closure	NA	+	NA	NA
	Vacuum-assisted closure	NA	+	NA	NA
**Wound area/size reduction** (MAs: NA; non-MAs: 2) [71,108]	Alginate dressings	NA	NA	–	NA
	Foam dressings	NA	NA	–	NA
**Time to healing or rate of healing** (MAs: NA; non-MAs: 5) [68,71,96,108,109]	Alginate dressings	NA	NA	+/−	NA
	Aloe vera dermal gel (topical)	NA	NA	+/−	NA
	Dextranomer polysaccharide bead dressings	NA	NA	+/−	NA
	Foam dressings	NA	NA	–	NA
	Gauze + Aloe vera dressings	NA	NA	+/−	NA
	Honey (topical)	NA	NA		+
	Hydrocolloid dressings	NA	NA	–	NA
	Plaster casting	NA	NA	+	NA
	Polyurethane foam and sheets dressings	NA	NA	+/−	NA
	Silicone elastomer foam dressings and polyurethane foam dressings	NA	NA	–	NA
	Topical negative pressure	NA	NA	–	NA
**Ulcer healing** (MAs: NA; non-MAs: 2) [71,108]	Dextranomer polysaccharide bead dressings	NA	NA	+/−	NA
	Polyurethane foam dressings	NA	NA	+/−	NA

*At least one systematic review with meta-analysis and AMSTAR score ≥8.

**At least one systematic review without meta-analysis and AMSTAR score ≥8.

+ Effective (statistically significant difference between interventions and comparators); – No difference (no statistically significant difference between interventions and comparators); +/− Unknown (conflicting evidence between meta-analysis or indeterminate results); NA, No studies available; MA, Meta-analysis.

### Methodological quality appraisal

**Figure 2:**
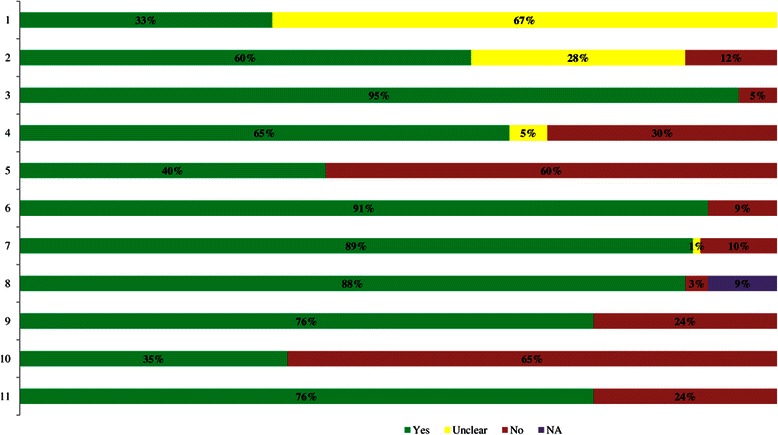
**AMSTAR methodological quality results.** NA, Not applicable. 1. *A priori* design. 2. Duplicate selection/DA. 3. Literature search. 4. Publication status. 5. List of studies. 6. Study characteristics. 7. Quality assessed. 8. Quality used. 9. Methods appropriate. 10. Publication bias assessed. 11. Conflicts stated.

Almost half (45%) of the systematic reviews were deemed high quality with an AMSTAR score ≥8 out of a possible 11 (Figure 2; Additional file 5). Many systematic reviews did not provide a list of excluded studies from screening potentially relevant full-text articles (60%) or address publication bias (65%). Conversely, 95% searched at least two electronic databases, 91% provided the characteristics of included studies, and 89% adequately used the quality appraisal results in formulating conclusions. Half of the systematic reviews including a meta-analysis had an AMSTAR score ≥8, and 40% of the systematic reviews without a meta-analysis had an AMSTAR score ≥8.

### Outcome results for venous leg ulcers

#### Wound area/size reduction

Two systematic reviews including two meta-analyses examined venous leg ulcer area/size reduction [56] (Table 2; Additional file 4), one of which had an AMSTAR score ≥8 [36]. Topical cadexomer iodine [36] and oral micronized purified flavonoid fraction [56] were more effective than placebo in each meta-analysis comparing these interventions.

#### Time to healing or rate of healing

Five systematic reviews including six meta-analyses [34,39,44,45,56] and one systematic review without a meta-analysis [77] (Table 2; Additional files 4 and 6) examined the time to healing for venous leg ulcers. Two of these had an AMSTAR score ≥8 [44,45].

Two systematic reviews (AMSTAR score ≥8) including two meta-analyses found that four-layer bandages were more effective than short stretch bandages [44] and compression systems [45]. One systematic review including two meta-analyses found conflicting results for bandages versus stockings [39]. Oral micronized purified flavonoid fraction was more effective than placebo in one meta-analysis [56].

#### Ulcer healing

Five systematic reviews including eight meta-analyses [29,30,39,51,62] and five systematic reviews without a meta-analysis [72,73,77,89,103] (Table 2; Additional files 4 and 6) examined venous leg ulcer healing. Four of these had an AMSTAR score ≥8 [30,62,73,89].

Elastic high compression was more effective than multi-layer inelastic compression in a systematic review (AMSTAR score ≥8) with a meta-analysis [62] and stockings were more effective than bandages in another meta-analysis [39]. Tissue engineered skin was more effective than dressings in one meta-analysis [51], topical granulocyte-macrophage colony stimulating factor was more effective than placebo in another meta-analysis [29], and oral pentoxifylline was more effective with (or without) compression than placebo in another meta-analysis (AMSTAR score ≥8) [30].

#### Proportion of patients with healed wounds

Nineteen systematic reviews with 39 meta-analyses [21,23,25,30,31,36,40,45,46,48,52,53,55,58,62,64-67] and two systematic reviews without a meta-analysis [77,102] (Table 2; Additional files 4 and 6) examined the proportion of patients with healed venous leg ulcers. Twelve of these had an AMSTAR score ≥8 [23,25,30,31,36,40,45,52,55,62,64,67].

Multi-layered, high compression bandages reduced ulcers compared with single layer bandages in two meta-analyses [62,67]. Elastic high compression was more effective than inelastic bandages [45] and versus inelastic compression bandages (AMSTAR score ≥8) [67] in two meta-analyses. Intermittent pneumatic compression was more effective than compression stockings or Unna’s boot in one meta-analysis (AMSTAR score ≥8) [67] and high compression stockings were more effective than compression bandages in another meta-analysis (AMSTAR score ≥8) [62]. Two-layer stockings were more effective than short-stretch bandages in another meta-analysis [44]. Ultrasound was more effective than no ultrasound in one meta-analysis [65] and cleaning wounds with cadexomer iodine plus compression therapy was more effective than standard care in another meta-analysis (AMSTAR score ≥8) [36]. Skin replacement therapy was more effective than standard compression therapy in one meta-analysis [46] and oral pentoxifylline with (or without) compression was more effective than placebo in two meta-analyses (AMSTAR score ≥8) [30]. Periulcer injection with granulocyte-macrophage colony stimulating factor was more effective than control in another meta-analysis [53].

### Outcome results for mixed arterial/venous leg ulcers

#### Wound area/size reduction

Two systematic reviews including three meta-analyses examined wound area/size reduction for mixed arterial/venous leg ulcers [34,65] (Table 3; Additional file 4). None had an AMSTAR score ≥8. One meta-analysis found topical silver and silver dressings more effective than placebo or conservative wound care or non-silver therapies [34] and another that ultrasound was more effective than standard treatment or placebo [65].

#### Time to healing or rate of healing

Four systematic reviews including five meta-analyses examined the time to healing for mixed arterial/venous leg ulcers [45,49,55,56] (Table 3; Additional file 4). Two had an AMSTAR score ≥8 [45,55]. One meta-analysis found topical negative pressure more effective than conventional therapy [49], a second found oral micronized purified flavonoid more effective than placebo or standard compression [56], and a third found that four-layer bandage was more effective than compression systems [45].

#### Ulcer healing

Six systematic reviews without a meta-analysis examined ulcer healing for mixed arterial/venous leg ulcers [80,82-84,101,110] (Table 3; Additional file 6). Two had an AMSTAR score ≥8 [80,84]. Details of these study results can be found in Additional file 6.

#### Proportion of patients with healed wounds

Two systematic reviews including three meta-analyses examined the proportion of patients with healed mixed arterial/venous wounds [49,50] (Table 3; Additional file 4). None had an AMSTAR score ≥8. Topical negative pressure was more effective than conventional therapy in one meta-analysis [49].

### Outcome results for diabetic foot/leg ulcers

The following outcome results were only reported in systematic reviews without meta-analysis: wound area/size reduction (n = 2) [79,88] and time to healing or rate of healing (n = 3) [79,88,95]. Details of these study results can be found in Additional file 6.

#### Ulcer healing

Three systematic reviews including four meta-analyses [24,35,57] and 10 systematic reviews without a meta-analysis [69,70,74,79,81,86,88,93,98,112] (Table 4*;* Additional files 4 and 6) examined healing improvements of diabetic foot/leg ulcers. Three had an AMSTAR score ≥8 [24,74,98].

One meta-analysis found that subcutaneous or intravenous granulocyte colony-stimulating factor plus oral or intravenous antibiotics was more effective than control (high quality) [24]. Another meta-analysis found that Chinese herbal medicine (see Table 4 and Additional file 4 for the exact preparation) plus unspecified standard therapy was more effective than standard therapy alone [35].

#### No healing improvement or non-healed wounds

Three systematic reviews including five meta-analyses [22,33,35] examined no healing improvement for diabetic wounds (Table 4; Additional file 4). Two of these had an AMSTAR score ≥8 [22,33].

Hyaluronic acid derivative was more effective than standard care in one meta-analysis (AMSTAR score ≥8) [22]. Low frequency low intensity noncontact ultrasound was more effective than sharps debridement in two meta-analyses (AMSTAR score ≥8) [33] and Chinese herbal medicine (see Additional file 4 for the exact preparation) plus unspecified standard therapy was more effective than standard therapy alone in another meta-analysis [35].

#### Proportion of patients with healed wounds

Fifteen systematic reviews including 18 meta-analyses [17-20,22,24,27,28,35,38,46,47,57-59] (Table 4; Additional file 4) examined the proportion of patients with healed diabetic foot and leg ulcers. Eight of these had an AMSTAR score ≥8 [17,18,20,22,24,27,28,59].

Hydrogel dressings were more effective than basic wound dressings, basic contact dressings, and gauze in three meta-analyses (two with an AMSTAR score ≥8) [17,19,28]. Artificial skin grafts were more effective than usual care in one meta-analysis [58] and skin replacement therapy was more effective than usual care in another [47]. Chinese herbal medicine (see Additional file 4 for the exact preparation) plus unspecified standard therapy was more effective than standard therapy alone in a meta-analysis [35] and subcutaneous or intravenous granulocyte colony-stimulating factor was more effective than usual care in another (AMSTAR score ≥8) [24]. Finally, a meta-analysis found platelet-rich plasma more effective than control [38].

### Outcome results for pressure ulcers

The following outcome results were only reported in systematic reviews without meta-analysis: wound area/size reduction (n = 3) [78,87,94] and time to healing or rate of healing (n = 3) [68,78,94]. Details of these study results can be found in Additional file 6.

#### Ulcer healing

Five systematic reviews reporting on eight meta-analyses [16,43,60,64,62] and 11 systematic reviews without a meta-analysis [75,76,78,85,90,92,94,97,99,104,106] (Table 5; Additional files 4 and 6) focused on pressure ulcer healing. Of these, eight had an AMSTAR score ≥8 [16,60,62,64,75,76,90,106].

Hydrocolloid dressings were more effective than usual care in a meta-analysis (AMSTAR score ≥8) [64], electrotherapy was more effective than sham therapy in another meta-analysis (AMSTAR score ≥8) [62], and air-fluidized beds were more effective than standard care or conventional mattresses in three meta-analyses [43,60,125]. In addition, alternate foam mattresses were more effective than standard foam mattresses in a meta-analysis (AMSTAR score ≥8) [60].

#### Proportion of patients with healed wounds

Three systematic reviews including 25 meta-analyses [43,54,62] and two systematic reviews without a meta-analysis [78,94] (Table 5; Additional files 4 and 6) examined the proportion of patients with healed pressure ulcers. Only one had an AMSTAR score ≥8 [62].

One meta-analysis found that hydrocolloid dressings were more effective than traditional dressings and another that hydrogel dressings were more effective than hydrocolloid dressings [43]. Different brands of alternating pressure mattresses were more effective than others in a meta-analysis [43].

### Outcome results for mixed complex wounds (unspecified)

#### Wound area/size reduction

Four systematic reviews including five meta-analyses examined the area/size reduction of mixed complex wounds (unspecified) [21,32,41,42] (Table 6; Additional file 4). Two had an AMSTAR score ≥8 [41,42]. Silver-impregnated dressings were more effective than dressings not containing silver in a meta-analysis (AMSTAR score ≥8) [41]. Topical negative pressure was more effective than standard wound care in another meta-analysis [32].

#### Ulcer healing

One systematic review including a meta-analysis [63] and five systematic reviews without a meta-analysis [91,100,105,107,111] (Table 6; Additional files 4 and 6) examined ulcer healing for mixed complex wounds. Three had an AMSTAR score ≥8 [91,107,111].

#### Proportion of patients with healed wounds

Five systematic reviews including 10 meta-analyses [21,55,58,61,62] (Table 6; Additional file 4) examined the proportion of patients with healed complex wounds. Two had an AMSTAR score ≥8 [55,62]. Hydrocolloid dressings were more effective than conventional dressings in a meta-analysis [61] and ultrasound was more effective than no ultrasound in another (AMSTAR score ≥8) [62]. In addition, artificial skin grafts were more effective than standard care in three meta-analyses [58].

### Outcome results for surgical wound infections

The following outcome results were only reported in systematic reviews without meta-analysis: wound area/size reduction (n = 2) [71,108], time to healing or rate of healing (n = 5) [68,71,96,108,109], and ulcer healing (n = 2) [71,108]. Details of these study results can be found in Additional file 6.

#### Proportion of patients with healed wounds

One systematic review including a meta-analysis (AMSTAR <8) found that topical negative pressure was more effective than standard treatment [37] (Table 7; Additional file 4).

#### Length of hospital stay

One systematic review and meta-analysis (AMSTAR <8) found that vacuum-assisted closure was more effective than conventional therapy for decreasing the length of hospital stay associated with surgical wound infections [26] (Table 7; Additional file 4).

## Discussion

We conducted a comprehensive overview of systematic reviews to identify optimal interventions for complex wounds. Data from 99 systematic reviews were scrutinized and interventions that are likely optimal were identified. These reviews examined numerous treatments and comparators and used different outcomes to assess effectiveness. Frequently, treatments considered as the intervention in one review were administered to the control in another. This renders the interpretation of our findings difficult.

We found that some interventions are likely to be effective based on data from systematic reviews including a meta-analysis with an AMSTAR score ≥8. For venous leg ulcers, four-layer bandages [44,45], elastic high compression [62], oral pentoxifylline with (or without) compression [30], compression bandages (multi-layer, elastic) [62,67], high compression [62] or multi-layer stockings [45], and wound cleansing with cadexomer iodine plus compression therapy [36] were effective compared with usual care. Only four-layer bandages [45] were effective in healing mixed arterial/venous leg ulcers versus compression systems. For diabetic foot/leg ulcers, subcutaneous or intravenous granulocyte colony-stimulating factor [24], hyaluronic acid derivative [22], low frequency, low intensity noncontact ultrasound [33], and hydrogel dressings [17,28] were effective interventions compared with usual care. For pressure ulcers, hydrocolloid dressings [64], electrotherapy [62], air-fluidized beds [60], and alternate foam mattresses [60] were effective compared with usual care. For mixed complex wounds, silver dressings [41] and ultrasound [62] were found to be more effective than no treatment. Finally, effective treatments were not identified for surgical wound infections amongst those with an AMSTAR score ≥8 including a meta-analysis. It is important to note that many of these interventions had conflicting results versus other comparators or were based on meta-analyses including few studies with a small number of patients. As such, these results should be interpreted with caution.

There are some limitations to our overview of systematic reviews. An inherent drawback of including systematic reviews is that the studies included in each of the reviews will have been published well before the search date. The inclusion of close to 100 systematic reviews, however, provides a breadth of information that is unlikely to significantly change with the inclusion of recently published studies. Although we appraised the methodological quality of the included reviews, we did not assess the risk of bias in the included studies in the systematic reviews. This is because a risk of bias tool for systematic reviews currently does not exist, but we are aware of one being developed by the Cochrane Collaboration [126]. Furthermore, we only included systematic reviews that were disseminated in English due to resource constraints. However, we attempted contacting authors to receive English translations. In addition, although we included five unpublished systematic reviews, we attempted to obtain unpublished data from a further 10 systematic reviews that were available as conference abstracts, yet we didn’t receive a response from the review authors. As such, our findings are likely representative of published literature written in English. Since we did not conduct a meta-analysis, we were unable to formally test for publication bias.

Our results suggest the need for a network meta-analysis [127], given the numerous interventions and comparators available and examined across the literature. Policy-makers focus their decisions at the systems level so require information on all treatment comparisons available. Patients and their healthcare providers need to know if the treatment they are prescribed or recommending is the most effective and safest compared with all others available. Conducting a high-quality, comprehensive systematic review and network meta-analysis is the only feasible tool available to examine multiple treatment comparisons. As the healthcare system shifts towards more complex problems and a resource-scarce environment, systematic reviews and meta-analysis of only one treatment comparison become obsolete. This is indeed the case for complex wound care interventions; despite the availability of almost 100 systematic reviews, optimal management is still unclear. Network meta-analysis also allows the ranking of all treatments for each effectiveness and safety outcome examined, making it a particularly attractive tool for decision-makers.

Almost half of the included systematic reviews were rated as being of high methodological quality according to the AMSTAR tool [13]. Consistent methodological shortcomings include not using a protocol to guide their conduct, not including a list of excluded studies at the full-text level of screening, and not addressing or referring to publication bias. Results reported in systematic reviews with higher scores on the AMSTAR tool are likely the most reliable. Furthermore, some studies only gave wound care patients two days of treatment or followed patients for two days. The utility of these short studies is questionable and studies of longer duration are recommended.

## Conclusions

In conclusion, our results confirm that there are numerous interventions that can be utilized for patients with complex wounds. However, few treatments were consistently effective throughout the literature. Clinicians and patients can use our results as a guide towards tailoring effective treatment according to type of wound. Planned future analysis of this data through network meta-analysis will further assist decision-makers as it will permit multiple treatment comparisons as well as the ranking of the effectiveness of all available wound care interventions.

## Additional files

Additional file 1:
**Classification of wound care interventions and comparators.** Lists the interventions and the corresponding comparators for each wound care treatment/comparator identified in our review. Additional file 2:
**MEDLINE search strategy.** Lists MEDLINE search terms. Additional file 3:
**Systematic review characteristics.** Lists the characteristics of studies included in the overview of reviews. Additional file 4:
**Meta-analysis results.** Provides relevant findings from the included reviews with a meta-analysis. Additional file 5:
**AMSTAR results for each systematic review.** Results of the AMSTAR quality assessment for included reviews. Additional file 6:
**Non-meta-analysis results.** Provides relevant findings from the included reviews without a meta-analysis.

## Abbreviation

AMSTAR: Assessment of Multiple Systematic Reviews

## References

[1] Akagi I, Furukawa K, Miyashita M, Kiyama T, Matsuda A, Nomura T (2012). Surgical wound management made easier and more cost-effective. Oncol Lett..

[2] Ferreira MC, Tuma P, Carvalho VF, Kamamoto F (2006). Complex wounds. Clinics (Sao Paulo)..

[3] Stansby G, Avital L, Jones K, Marsden G, Guideline Development Group (2014). Prevention and management of pressure ulcers in primary and secondary care: summary of NICE guidance. BMJ.

[4] Adderley U, Smith R (2007). Topical agents and dressings for fungating wounds. Cochrane Database Syst Rev..

[5] Abidia A, Laden G, Kuhan G, Johnson BF, Wilkinson AR, Renwick PM (2003). The role of hyperbaric oxygen therapy in ischaemic diabetic lower extremity ulcers: a double-blind randomised-controlled trial. Eur J Vasc Endovasc Surg..

[6] Apelqvist J, Ragnarson TG (1996). Cavity foot ulcers in diabetic patients: a comparative study of cadexomer iodine ointment and standard treatment. An economic analysis alongside a clinical trial. Acta Derm Venereol.

[7] Harding KG, Morris HL, Patel GK (2002). Science, medicine and the future: healing chronic wounds. BMJ..

[8] Brolmann FE, Ubbink DT, Nelson EA, Munte K, van der Horst CM, Vermeulen H (2012). Evidence-based decisions for local and systemic wound care. Br J Surg..

[9] Ubbink DT, Santema TB, Stoekenbroek RM (2014). Systemic wound care: a meta-review of cochrane systematic reviews. Surg Technol Int..

[10] Higgins JPT, Green S. Cochrane Handbook for Systematic Reviews of Interventions. Version 5.1.0 [updated March 2011]. The Cochrane Collaboration, 2011. www.cochrane-handbook.org.

[11] National Institute for Health Research (NIH). PROSPERO database http://www.crd.york.ac.uk/Prospero/.

[12] Sampson M, McGowan J, Cogo E, Grimshaw J, Moher D, Lefebvre C (2009). An evidence-based practice guideline for the peer review of electronic search strategies. J Clin Epidemiol..

[13] Shea BJ, Hamel C, Wells GA, Bouter LM, Kristjansson E, Grimshaw J (2009). AMSTAR is a reliable and valid measurement tool to assess the methodological quality of systematic reviews. J Clin Epidemiol..

[14] Shea BJ, Bouter LM, Peterson J, Boers M, Andersson N, Ortiz Z (2007). External validation of a measurement tool to assess systematic reviews (AMSTAR). PLoS One..

[15] Tricco AC, Tetzlaff J, Pham B, Brehaut J, Moher D (2009). Non-Cochrane vs. Cochrane reviews were twice as likely to have positive conclusion statements: cross-sectional study. J Clin Epidemiol.

[16] Stratton RJ, Ek AC, Engfer M, Moore Z, Rigby P, Wolfe R (2005). Enteral nutritional support in prevention and treatment of pressure ulcers: a systematic review and meta-analysis. Ageing Res Rev..

[17] Dumville JC, Deshpande S, O’Meara S, Speak K (2012). Hydrocolloid dressings for healing diabetic foot ulcers. Cochrane Database Syst Rev..

[18] Dumville JC, O’Meara S, Deshpande S, Speak K (2012). Alginate dressings for healing diabetic foot ulcers. Cochrane Database Syst Rev..

[19] Edwards J, Stapley S (2012). Debridement of diabetic foot ulcers. Cochrane Database Syst Rev..

[20] Kranke P, Bennett MH, Martyn-St James M, Schnabel A, Debus SE (2012). Hyperbaric oxygen therapy for chronic wounds. Cochrane Database Syst Rev..

[21] Martinez-Zapata MJ, Marti-Carvajal AJ, Sola I, Exposito JA, Bolibar I, Rodriguez L (2012). Autologous platelet-rich plasma for treating chronic wounds. Cochrane Database Syst Rev..

[22] Voigt J, Driver VR (2012). Hyaluronic acid derivatives and their healing effect on burns, epithelial surgical wounds, and chronic wounds: a systematic review and meta-analysis of randomized controlled trials. Wound Repair Regen..

[23] Wilkinson EA (2012). Oral zinc for arterial and venous leg ulcers. Cochrane Database Syst Rev..

[24] Cruciani M, Lipsky BA, Mengoli C, de Lalla F (2011). Granulocyte-colony stimulating factors as adjunctive therapy for diabetic foot infections. Cochrane Database Syst Rev..

[25] Cullum N, Al-Kurdi D, Bell-Syer SEM (2011). Therapeutic ultrasound for venous leg ulcers. Cochrane Database Syst Rev..

[26] Damiani G, Pinnarelli L, Sommella L, Tocco MP, Marvulli M, Magrini P (2011). Vacuum-assisted closure therapy for patients with infected sternal wounds: a meta-analysis of current evidence. J Plast Reconstr Aesthet Surg..

[27] Dumville JC, Deshpande S, O’Meara S, Speak K (2011). Foam dressings for healing diabetic foot ulcers. Cochrane Database Syst Rev..

[28] Dumville JC, O’Meara S, Deshpande S, Speak K (2011). Hydrogel dressings for healing diabetic foot ulcers. Cochrane Database Syst Rev..

[29] Hu X, Sun H, Han C, Wang X, Yu W (2011). Topically applied rhGM-CSF for the wound healing: a systematic review. Burns..

[30] Jull AB, Arroll B, Parag V, Waters J (2011). Pentoxifylline for treating venous leg ulcers. Cochrane Database Syst Rev..

[31] Nelson EA, Mani R, Thomas K, Vowden K (2011). Intermittent pneumatic compression for treating venous leg ulcers. Cochrane Database Syst Rev..

[32] Suissa D, Danino A, Nikolis A. Negative-pressure therapy versus standard wound care: a meta-analysis of randomized trials. Plast Reconstr Surg. 2011;128:498e–503e. 10.1097/PRS.0b013e31822b675c 22030509

[33] Voigt J, Wendelken M, Driver V, Alvarez OM (2011). Low-frequency ultrasound (20–40 kHz) as an adjunctive therapy for chronic wound healing: a systematic review of the literature and meta-analysis of eight randomized controlled trials. Int J Low Extrem Wounds..

[34] Carter MJ, Tingley-Kelley K, Warriner RA (2010). Silver treatments and silver-impregnated dressings for the healing of leg wounds and ulcers: a systematic review and meta-analysis. J Am Acad Dermatol..

[35] Chen M, Zheng H, Yin LP, Xie CG (2010). Is oral administration of Chinese herbal medicine effective and safe as an adjunctive therapy for managing diabetic foot ulcers? A systematic review and meta-analysis. J Altern Complement Med..

[36] O’Meara S, Al-Kurdi D, Ologun Y, Ovington LG (2010). Antibiotics and antiseptics for venous leg ulcers. Cochrane Database Syst Rev..

[37] Pan A, Cauda R, Concia E, Esposito S, Sganga G, Stefani S (2010). Consensus document on controversial issues in the treatment of complicated skin and skin-structure infections. Int J Infect Dis..

[38] Villela DL, Santos VL (2010). Evidence on the use of platelet-rich plasma for diabetic ulcer: a systematic review. Growth Factors..

[39] Amsler F, Willenberg T, Blattler W (2009). In search of optimal compression therapy for venous leg ulcers: a meta-analysis of studies comparing diverse [corrected] bandages with specifically designed stockings. J Vasc Surg..

[40] Jull AB, Rodgers A, Walker N (2009). Honey as a topical treatment for wounds. Cochrane Database Syst Rev..

[41] Lo SF, Chang CJ, Hu WY, Hayter M, Chang YT (2009). The effectiveness of silver-releasing dressings in the management of non-healing chronic wounds: a meta-analysis. J Clin Nurs..

[42] Martinez-Zapata MJ, Marti-Carvajal A, Sola I, Bolibar I, Angel Exposito J, Rodriguez L (2009). Efficacy and safety of the use of autologous plasma rich in platelets for tissue regeneration: a systematic review. Transfusion (Paris)..

[43] Health Quality Ontario. Management of chronic pressure ulcers: an evidence-based analysis. Ont Health Technol Assess Ser. 2009;9(3):1–203. PMC3377577 23074533

[44] O’Meara S, Tierney J, Cullum N, Bland JM, Franks PJ, Mole T (2009). Four layer bandage compared with short stretch bandage for venous leg ulcers: systematic review and meta-analysis of randomised controlled trials with data from individual patients. BMJ..

[45] O’Meara S, Cullum NA, Nelson EA (2009). Compression for venous leg ulcers. Cochrane Database Syst Rev..

[46] Barber C, Watt A, Pham C, Humphreys K, Penington A, Mutimer K (2008). Influence of bioengineered skin substitutes on diabetic foot ulcer and venous leg ulcer outcomes. J Wound Care..

[47] Blozik E, Scherer M (2008). Skin replacement therapies for diabetic foot ulcers: systematic review and meta-analysis. Diabetes Care..

[48] Flemming K, Cullum NA (2008). Laser therapy for venous leg ulcers. Cochrane Database Syst Rev..

[49] Sadat U, Chang G, Noorani A, Walsh SR, Hayes PD, Varty K (2008). Efficacy of TNP on lower limb wounds: a meta-analysis. J Wound Care..

[50] Chambers H, Dumville JC, Cullum N (2007). Silver treatments for leg ulcers: a systematic review. Wound Repair Regen..

[51] Jones JE, Nelson EA (2007). Skin grafting for venous leg ulcers. Cochrane Database Syst Rev..

[52] Palfreyman S, Nelson EA, Michaels JA (2007). Dressings for venous leg ulcers: systematic review and meta-analysis. BMJ..

[53] O’Donnell TF, Lau J (2006). A systematic review of randomized controlled trials of wound dressings for chronic venous ulcer. J Vasc Surg..

[54] Sari BA, Flemming K, Cullum NA, Wollina U (2006). Therapeutic ultrasound for pressure ulcers. Cochrane Database Syst Rev..

[55] Bouza C, Munoz A, Amate JM (2005). Efficacy of modern dressings in the treatment of leg ulcers: a systematic review. Wound Repair Regen..

[56] Coleridge-Smith P, Lok C, Ramelet AA (2005). Venous leg ulcer: a meta-analysis of adjunctive therapy with micronized purified flavonoid fraction. Eur J Vasc Endovasc Surg..

[57] Cruciani M, Lipsky BA, Mengoli C, de Lalla F (2005). Are granulocyte colony-stimulating factors beneficial in treating diabetic foot infections?. A meta-analysis. Diabetes Care..

[58] Ho C, Tran K, Hux M, Sibbald G, Campbell K. Artificial skin grafts in chronic wound care: a meta-analysis of clinical efficacy and a review of cost-effectiveness. In: Technology Report no 52. Ottawa: Canadian Coordinating Office for Health Technology Assessment; 2005.

[59] Roeckl-Wiedmann I, Bennett M, Kranke P (2005). Systematic review of hyperbaric oxygen in the management of chronic wounds. Br J Surg..

[60] Cullum N, Deeks J, Sheldon TA, Song F, Fletcher AW (2004). Beds, mattresses and cushions for pressure sore prevention and treatment. Cochrane Database Syst Rev..

[61] Singh A, Halder S, Menon GR, Chumber S, Misra MC, Sharma LK (2004). Meta-analysis of randomized controlled trials on hydrocolloid occlusive dressing versus conventional gauze dressing in the healing of chronic wounds. Asian J Surg..

[62] Cullum N, Nelson EA, Flemming K, Sheldon T (2001). Systematic reviews of wound care management: (5) beds; (6) compression; (7) laser therapy, therapeutic ultrasound, electrotherapy and electromagnetic therapy. Health Technol Assess..

[63] Lucas C, Stanborough RW, Freeman CL, De Haan RJ (2000). Efficacy of low-level laser therapy on wound healing in human subjects: a systematic review. Lasers Med Sci..

[64] Bradley M, Cullum N, Nelson EA, Petticrew M, Sheldon T, Torgerson D (1999). Systematic reviews of wound care management: (2). Dressings and topical agents used in the healing of chronic wounds. Health Technol Assess.

[65] Johannsen F, Gam AN, Karlsmark T (1998). Ultrasound therapy in chronic leg ulceration: a meta-analysis. Wound Repair Regen..

[66] Palfreyman SJ, Lochiel R, Michaels JA (1998). A systematic review of compression therapy for venous leg ulcers. Vasc Med..

[67] Fletcher A, Cullum N, Sheldon TA (1997). A systematic review of compression treatment for venous leg ulcers. BMJ..

[68] Dat AD, Poon F, Pham KB, Doust J (2012). Aloe vera for treating acute and chronic wounds. Cochrane Database Syst Rev..

[69] Hinchliffe RJ, Andros G, Apelqvist J, Bakker K, Friederichs S, Lammer J (2012). A systematic review of the effectiveness of revascularization of the ulcerated foot in patients with diabetes and peripheral arterial disease. Diabetes Metab Res Rev..

[70] Peters EJ, Lipsky BA, Berendt AR, Embil JM, Lavery LA, Senneville E (2012). A systematic review of the effectiveness of interventions in the management of infection in the diabetic foot. Diabetes Metab Res Rev..

[71] Vermeulen H, Ubbink DT, Goossens A, de Vos R, Legemate DA, Westerbos SJ (2012). Dressings and topical agents for surgical wounds healing by secondary intention. Cochrane Database Syst Rev..

[72] Zarchi K, Jemec GB (2012). The efficacy of maggot debridement therapy-a review of comparative clinical trials. Int Wound J..

[73] Aziz Z, Cullum NA, Flemming K (2011). Electromagnetic therapy for treating venous leg ulcers. Cochrane Database Syst Rev..

[74] Lima AF, Costa LB, Silva JL, Maia MB, Ximenes EC (2011). Interventions for wound healing among diabetic patients infected with Staphylococcus aureus: a systematic review. Sao Paulo Med J..

[75] McGinnis E, Stubbs N (2011). Pressure-relieving devices for treating heel pressure ulcers. Cochrane Database Syst Rev..

[76] McInnes E, Dumville JC, Jammali-Blasi A, Bell-Syer SE (2011). Support surfaces for treating pressure ulcers. Cochrane Database Syst Rev..

[77] Nelson EA. Venous leg ulcers. BMJ Clin Evid. 2011;2011:pii:1902. PMC3275133 22189344

[78] Reddy M. Pressure ulcers. BMJ Clin Evid. 2011;2011:pii:1901. PMC3217823 21524319

[79] Hunt DL. Diabetes: foot ulcers and amputations. BMJ Clin Evid. 2009;2009:pii:0602. PMC2907821 19445774

[80] Vermeulen H, van Hattem JM, Storm-Versloot MN, Ubbink DT, Westerbos SJ (2010). Topical silver for treating infected wounds. Cochrane Database Syst Rev..

[81] Xie X, McGregor M, Dendukuri N (2010). The clinical effectiveness of negative pressure wound therapy: a systematic review. J Wound Care..

[82] McGaughey H, Dhamija S, Oliver L, Porter-Armstrong A, McDonough S (2009). Pulsed electromagnetic energy in management of chronic wounds: a systematic review. Phys Ther Rev..

[83] Health Quality Ontario. Community-based care for chronic wound management: an evidence-based analysis. Ont Health Technol Assess Ser. 2009, 9(18):1–24. PMC3377537 23074522

[84] Nelson EA, Bradley MD (2009). Dressings and topical agents for arterial leg ulcers. Cochrane Database Syst Rev..

[85] Ramundo J, Gray M (2009). Collagenase for enzymatic debridement: a systematic review. J Wound Ostomy Continence Nurs..

[86] Roukis TS, Schade VL (2009). Percutaneous flexor tenotomy for treatment of neuropathic toe ulceration secondary to toe contracture in persons with diabetes: a systematic review. J Foot Ankle Surg..

[87] Heyneman A, Beele H, Vanderwee K, Defloor T (2008). A systematic review of the use of hydrocolloids in the treatment of pressure ulcers. J Clin Nurs..

[88] Hinchliffe RJ, Valk GD, Apelqvist J, Armstrong DG, Bakker K, Game FL (2008). A systematic review of the effectiveness of interventions to enhance the healing of chronic ulcers of the foot in diabetes. Diabetes Metab Res Rev..

[89] Howard DP, Howard A, Kothari A, Wales L, Guest M, Davies AH (2008). The role of superficial venous surgery in the management of venous ulcers: a systematic review. Eur J Vasc Endovasc Surg..

[90] Langer G, Knerr A, Kuss O, Behrens J, Schlomer GJ (2008). Nutritional interventions for preventing and treating pressure ulcers. Cochrane Database Syst Rev..

[91] Lo SF, Hayter M, Chang CJ, Hu WY, Lee LL (2008). A systematic review of silver-releasing dressings in the management of infected chronic wounds. J Clin Nurs..

[92] Moore Z, Cowman S (2008). A systematic review of wound cleansing for pressure ulcers. J Clin Nurs..

[93] Noble-Bell G, Forbes A (2008). A systematic review of the effectiveness of negative pressure wound therapy in the management of diabetes foot ulcers. Int Wound J..

[94] Reddy M, Gill SS, Kalkar SR, Wu W, Anderson PJ, Rochon PA (2008). Treatment of pressure ulcers: a systematic review. JAMA..

[95] Ubbink DT, Westerbos SJ, Evans D, Land L, Vermeulen H (2008). Topical negative pressure for treating chronic wounds. Cochrane Database Syst Rev..

[96] Ubbink DT, Westerbos SJ, Nelson EA, Vermeulen H (2008). A systematic review of topical negative pressure therapy for acute and chronic wounds. Br J Surg..

[97] Van den Boogaard M, Laat E, Spauwen P, Schoonhoven L (2008). The effectiveness of topical negative pressure in the treatment of pressure ulcers: a literature review. Eur J Plast Surg..

[98] Nelson EA, O’Meara S, Craig D, Iglesias C, Golder S, Dalton J, et al. A series of systematic reviews to inform a decision analysis for sampling and treating infected diabetic foot ulcers. Health Technol Assess. 2006;10:iii-iv–ix-x. 1–221. 10.3310/hta10120 16595081

[99] Bouza C, Saz Z, Munoz A, Amate JM (2005). Efficacy of advanced dressings in the treatment of pressure ulcers: a systematic review. J Wound Care..

[100] Schuren J, Becker A, Sibbald RG (2005). A liquid film-forming acrylate for peri-wound protection: a systematic review and meta-analysis (3 M Cavilon no-sting barrier film). Int Wound J..

[101] Mwipatayi BP, Angel D, Norrish J, Hamilton MJ, Scott A, Sieunarine K. The use of honey in chronic leg ulcers: a literature review. Primary Intention. 2004;12:107–8. 110–112.

[102] TenBrook JA, Iafrati MD, O’Donnell TF, Wolf MP, Hoffman SN, Pauker SG (2004). Systematic review of outcomes after surgical management of venous disease incorporating subfascial endoscopic perforator surgery. J Vasc Surg..

[103] Berliner E, Ozbilgin B, Zarin DA (2003). A systematic review of pneumatic compression for treatment of chronic venous insufficiency and venous ulcers. J Vasc Surg..

[104] Pham CT, Middleton P, Maddern G. Vacuum-assisted closure for the management of wounds: an accelerated systematic review. North Adelaide, S. Australia, Australia. Australian Safety and Efficacy Register of New Interventional Procedures (ASERNIP) - Surgical. ASERNIP-S Report; 37. http://www.surgeons.org/media/19301/VACaccelreview1203.pdf.

[105] Wang C, Schwaitzberg S, Berliner E, Zarin DA, Lau J (2003). Hyperbaric oxygen for treating wounds: a systematic review of the literature. Arch Surg..

[106] Emergency Care Research Institute (ECRI). Air-fluidized beds used for treatment of pressure ulcers in the home environment. https://www.ecri.org/Documents/EPC/Air-Fluidized_Beds_Used_for_Treatment_of_Pressure_Ulcers_in_the_Home_Environment.pdf 25905145

[107] Evans D, Land L (2001). Topical negative pressure for treating chronic wounds: a systematic review. Br J Plast Surg..

[108] Lewis R, Whiting P, ter Riet G, O’Meara S, Glanville J (2001). A rapid and systematic review of the clinical effectiveness and cost-effectiveness of debriding agents in treating surgical wounds healing by secondary intention. Health Technol Assess..

[109] Moore OA, Smith LA, Campbell F, Seers K, McQuay HJ, Moore RA (2001). Systematic review of the use of honey as a wound dressing. BMC Complement Altern Med..

[110] O’Meara SM, Cullum NA, Majid M, Sheldon TA (2001). Systematic review of antimicrobial agents used for chronic wounds. Br J Surg..

[111] Bradley M, Cullum N, Sheldon T. The debridement of chronic wounds: a systematic review. Health Technol Assess. 1999;3:iii–iv. 1–78. 10492854

[112] Mason J, O’Keeffe C, Hutchinson A, McIntosh A, Young R, Booth A (1999). A systematic review of foot ulcer in patients with Type 2 diabetes mellitus. II: treatment. Diabet Med..

[113] Vermeulen H, Ubbink DT, Goossens A, de Vos R, Legemate DA (2005). Systematic review of dressings and topical agents for surgical wounds healing by secondary intention. Br J Surg..

[114] Vermeulen H, Ubbink D, Semin-Goossens A, de Vos R, Legemate D. Dressings and topical agents for surgical wounds healing by secondary intention. Cochrane Database Syst Rev. 2004:CD003554. 10.1002/14651858.CD003554.pub2 PMC8407283 15106207

[115] Wilkinson E, Hawke C (1999). Zinc and chronic leg ulcers: a systematic review of oral zinc in the treatment of chronic leg ulcers. J Tissue Viability..

[116] Wilkinson EA, Hawke CI (1998). Does oral zinc aid the healing of chronic leg ulcers? A systematic literature review. Arch Dermatol..

[117] Aziz Z, Flemming K, Cullum NA, Olyaee MA (2010). Electromagnetic therapy for treating pressure ulcers. Cochrane Database Syst Rev..

[118] Jull AB, Arroll B, Parag V, Waters J (2007). Pentoxifylline for treating venous leg ulcers. Cochrane Database Syst Rev..

[119] Nelson EA, Jones J. Venous leg ulcers. BMJ Clin Evid. 2008;2008:pii:1902. PMC2908003 19445798

[120] Hunt D. Diabetes: foot ulcers and amputations. BMJ Clin Evid. 2009; 2009. PMC2907821 19445774

[121] O’Meara S, Al-Kurdi D, Ovington LG (2008). Antibiotics and antiseptics for venous leg ulcers. Cochrane Database Syst Rev..

[122] Nelson EA, Bradley MD (2006). Dressings and topical agents for arterial leg ulcers. Cochrane Database Syst Rev..

[123] Ramundo J, Gray M (2008). Enzymatic wound debridement. J Wound Ostomy Continence Nurs..

[124] O’Meara S, Cullum N, Majid M, Sheldon T (2000). Systematic reviews of wound care management: (3) antimicrobial agents for chronic wounds; (4) diabetic foot ulceration. Health Technol Assess..

[125] Cullum N, Petherick E. Pressure ulcers. BMJ Clin Evid. 2008;2008:pii:1901. PMC2907959 19450317

[126] University of Bristol. ROBIS tool. http://www.robis-tool.info/.

[127] Catala-Lopez F, Tobias A, Cameron C, Moher D, Hutton B (2014). Network meta-analysis for comparing treatment effects of multiple interventions: an introduction. Rheumatol Int.

