# Supplementary material for: Seeking effective interventions to treat complex wounds: an overview of systematic reviews
Source: BMC Med. 2015 Apr 22;13:89. doi: 10.1186/s12916-015-0288-5 (PMC4406332; doi:10.1186/s12916-015-0288-5)
Supplement: Additional file 1: — Classification of wound care interventions and comparators. Lists the interventions and the corresponding comparators for each wound care treatment/comparator identified in our review. [file 12916_2015_288_MOESM1_ESM.pdf]

Classification of wound care interventions and comparators	
**1. Bandages**	
Interventions<br>2-component (outer elastic)<br>4-layer bandage<br>Compression bandages<br>High compression (elastic, multilayer)<br>No-sting barrier film<br>Unna's boot	*Comparators*<br>2-component (outer elastic)<br>Compression (multi-layer, single layer, inelastic, elastic)<br>Paste bandage plus compression<br>Short stretch bandage (multi-layer, single layer)<br>Single-layer<br>Unna's boot
**2. Biologics**	
Interventions<br>Arginine-glycine-aspartic acid peptide matrix (topical)<br>Autologous platelet-rich plasma/platelet-rich plasma (topical)<br>Calcitonin gene-related peptide (topical)<br>Granulocyte-macrophage colony stimulating factor (subcutaneous, peri-ulcer injection)<br>Hyaluronic acid based<br>Nerve growth factor (topical)<br>Prostaglandin E1 (intravenous)<br>Protease-modulating matrix (topical)<br>Recombinant platelet derived/fibroblast growth factor (topical)<br>Stem cell therapy<br>Sulodexide (oral)<br>Systemic mesoglycan (intra-muscular, oral)<br>Thrombin-induced human platelet growth factor (topical)<br>Thromboxane α2 antagonists (oral)<br>Transforming growth factor beta (topical)	*Comparators*<br>Not applicable
**3. Complementary and alternative medicine**	
Interventions<br>Ayurevedic medicine (oral and topical)<br>Chinese herbal medicine (Bu-yang-huan-wu decoction, Tao-hong-si-wu decoction, Si-miao-yong-an decoction, Radix astragali, Rhizoma atractylodis, marcocephalae, Radix stephaniae tetrandrae, Radix Polygoni multifori, Radix Rehmanniae, Radix smilax china, Frusctus corni, Rhizoma dioscoreae, Cortex Moutan, Rhizoma alismatis, Rhizoma smilacis glabrae, Frutus schisandrae, Herba Siegesbeckiae, Draconis Sanguis, Lumbricus, Radix Ligustici, Chuanxiogm, Ramulus Cinnamomi Cassiae, She-xiang-huo-xue capsule)	*Comparators*<br>Not applicable
**4. Devices/adjuvant therapy**	
Interventions<br>Electrical stimulation<br>Electromagnetic therapy<br>Hyperbaric oxygen therapy<br>Laser therapy<br>Light therapy (monochromatic, UV, polarized)<br>Magnet and normothermic therapy<br>Topical negative pressure<br>Ultrasound (high frequency, low frequency)	*Comparators*<br>Sham device/adjuvant therapy
**5. Dressings**	
Interventions	*Comparators*

Alginate<br>Aloe vera<br>Aluminin hydroxide<br>Amino acid copolymer<br>Antibiotic<br>Cadexomer iodine<br>Cellulose<br>Collagen<br>Dextranomer<br>Dialysate<br>Foam<br>Hyaluronic acid-derived<br>Hydrocolloid<br>Hydrogel<br>Hydropolymer<br>Insulin<br>Live yeast derivative<br>Moist saline<br>Noncontact normothermic<br>Oxyquinoline<br>Phenytoin solution<br>Polyhydroxyethyl metahcrylate<br>Polysaccharide<br>Polyurethane<br>Radiant heat<br>Resin salve<br>Semelil gel<br>Silicone<br>Silver<br>Streptokinase-streptodornase<br>Vitamin A<br>Zinc oxide tape or salt spray	Absorbent acrylic<br>Acetic acid<br>Acetic acide<br>Alginate<br>Antiseptic agents<br>Biosynthetics<br>Bolster<br>Collagen<br>Copolymer membrane<br>Dextraonomer<br>Egg white<br>Eusol<br>Fibrinolysin<br>Foam<br>Honey<br>Hydrocolloid<br>Hydrogel<br>Lanolin<br>Low adherent<br>Papin-urea ointment<br>Paraffin gauze<br>Petrolateum<br>Polyurethane<br>Povidone-iodine or cadexomer-iodine<br>Radiant heat<br>Saline-moistened gauze<br>Silicone<br>Sodium hypochlorite<br>Sugar<br>Topical enzymes<br>Transparent film<br>Wet-to-moist gauze
**6. Nutritional supplementation**	
Interventions<br>Arginine<br>Ascorbic acid<br>Collagen protein hydrolysate<br>Disease-specific nutrition treatment<br>High-protein diet<br>Zinc sulphate	*Comparators*<br>Not applicable
**7. Other oral treatment**	
Interventions<br>Antibiotics (ciprofloxacin, trimethoprim, levamisole, amoxylcillin, clindamycin, oral, cefotoxin, metronidazole, gentamicin, Eusol pack, clindamycin, fluoroquinolone, rifampicin, amoxicillin/clavulanic acid, imipenem/cilastatin, cefazolin, ampicillin/sulbactam, linezolid, piperacillin/tazobactam, clindamycin hydrochloride)<br>Flavonoids<br>Ketanserin<br>Micronized purified flavonoid fraction (MPFF)<br>Pentoxifylline<br>Rutosides<br>Systemic ciprofloxacin<br>Zinc	*Comparators*<br>Antibiotics (unspecified)

**8. Other topical treatment**	
Interventions<br>Aloe vera<br>Antibiotics (polynoxilin, dimethyl sulfoxide, mupirocin)<br>Antimicrobial<br>Enzymatic agents<br>Honey<br>Honey<br>Hydrogel<br>Ketanserin<br>Ketanserin ointment (2%) (topical)<br>Lyophilized collagen<br>Mesoglycan<br>Phenytoin<br>Procaine<br>Silver<br>Topical sugar	*Comparators*<br>Disinfectants<br>Enzymatic agents<br>Hydrogel<br>Plant-based extract<br>Polyethylene glycol
**9. Skin replacement therapy**	
Interventions<br>Allografts (fresh, split thickness)<br>Applied freeze-dried keratinocyte lysate<br>Cryopreserved allografts<br>Cultured keratinocytes/epidermal/ allogenic bilayer<br>Fibroblast-derived<br>Tissue engineered skin	*Comparators*<br>Split thickness graft
**10. Stockings**	
Interventions<br>Casting (Total contact casting, plaster cast)<br>High-compression regimens<br>Intermittent pneumatic compression<br>Multilayer elastic system, multilayer elastomeric (or non-elastomeric)<br>Single-layer non-elastic system	*Comparators*<br>Compression<br>Intermittent pneumatic compression
**11. Support surfaces**	
Interventions<br>Air-fluidised bed/supports<br>Alternating pressure mattress<br>Foam mattress (alternative foam, specialized foam)<br>Low air-loss beds<br>Low-tech constant-low-pressure supports<br>Pressure off-loading (total contact or non-removable cast)<br>Pressure off-loading (felted foam)<br>Seat cushions<br>Therapeutic footwear	*Comparators*<br>Air mattress<br>Alternating pressure mattress<br>Conventional mattresses<br>Fluid mattress overlay<br>Foam mattress (standard foam, specialized foam)<br>Low-tech constant-low-pressure supports<br>Pressure off-loading (pressure-relief half shoe)<br>Usual footwear<br>Water mattress
**12. Surgery**	
Interventions<br>Early surgical intervention<br>Endovascular or open bypass revascularization surgery of an ulcerated foot<br>Percutaneous flexor tenotomy<br>Resection of the chronic wound<br>Subfascial endoscopic perforator surgery<br>Superficial vein surgery<br>Superficial venous surgery	*Comparators*<br>Not applicable

13. **Wound care programs**	
Interventions<br>Foot care clinic<br>Leg ulcer clinics<br>Patient education<br>Primary nurse delivery, clinician education, standardized treatment, team of community pharmacists and nurses	*Comparators*<br>Not applicable
14. **Wound cleansing**	
Interventions<br>Cadexomer iodine<br>Cadexomer iodine<br>Collagenase debridement (topical)<br>Larval therapy<br>Wound cleansing agents (collagenase, topical)<br>Wound cleansing (Maggot debridement therapy)<br>Wound cleansing agents (Dextranomer polysaccharide beads or paste, Cadexomer iodine polysaccharide beads or paste)<br>Wound cleansing agents (Dextranomer polysaccharide beads or paste, Cadexomer iodine polysaccharide beads or paste)<br>Wound cleansing agents (Dextranomer polysaccharide beads or paste, Cadexomer iodine polysaccharide beads or paste)<br>Larval Therapy<br>Topical agents (superoxidized water and soap, povidone iodine)<br>Wound cleansing (Sharp debridement)<br>Wound cleansing (saline spray containing aloe vera, silver chloride and decyl glucoside (Vulnopur), saline, whirlpool)<br>Wound cleansing (Collagenase enzymatic)<br>Wound cleansing (collegenase)	*Comparators*<br>Not applicable
