# Supplementary material for: Seeking effective interventions to treat complex wounds: an overview of systematic reviews
Source: BMC Med. 2015 Apr 22;13:89. doi: 10.1186/s12916-015-0288-5 (PMC4406332; doi:10.1186/s12916-015-0288-5)
Supplement: Additional file 3: — Systematic review characteristics. Lists the characteristics of studies included in the overview of reviews. [file 12916_2015_288_MOESM3_ESM.pdf]

Systematic Review Characteristics										
**Study**	**Country of conduct**	**No. studies**	**RCT Study design**	**Wound type(s)**	**Age category (years)**	**Patient Population**	**Comorbidities**	**Care setting**	**Range of treatment period**	**Range of follow up period**
Dat, 2012[68]	Australia	2	Yes	Pressure ulcers, infected surgical wounds	Any age	NR	NR	Hospital, clinic	Daily-10 weeks	<12 weeks
Dumville, 2012a[17]	UK	4	Yes	Diabetic foot/leg ulcers	18-80	Type I & II diabetics	NR	NR	Daily-24 weeks	8-35.7 weeks
Dumville, 2012b[18]	UK	6	Yes	Diabetic foot/leg ulcers	Adults	Type I & II diabetics	NR	NR	NR	4-12 weeks
Edwards, 2012[19]	UK	6	Yes	Diabetic foot/leg ulcers	NR	Type I & II diabetics	NR	Out-patients	NR	<16 weeks
Hinchliffe, 2012[69]	UK	49	No	Diabetic foot/leg ulcers	36-74	Type I & II diabetics	Cardiovascular, renal and cerebral vascular diseases	Hospital	NR	30 days-7 years
Kranke, 2012[20]	Germany	9	Yes	Diabetic foot/leg ulcers	NR	Type I & II diabetics, venous insufficiency	NR	NR	10 days-10 weeks	NR
Martinez-Zapata, 2012[21]	Spain	9	Yes	Mixed chronic wounds, venous leg ulcers	NR	NR	NR	Hospital	8-40 weeks	NR
Peters, 2012[70]	Netherlands	33	Yes	Diabetic foot/leg ulcers	≥18	Type I & II diabetics	Diabetes, skin and soft tissue infection, foot ulcers, post-surgical wounds, osteomyelitis, cellulitis	NR	5 days-9.7 weeks	NR
Vermeulen, 2012[71] (CR: Vermeulen,	Netherlands	13	Yes	Infected surgical wounds	>18	NR	NR	All settings	NR	NR

2005[113], Vermeulen, 2004[114])										
Voigt, 2012[22]	USA	10	Yes	Diabetic foot/leg ulcers	20-70	NR	Diabetic, cancer	NR	2-13 weeks	4 weeks-78 weeks
Wilkinson, 2012[23] (CR: Wilkinson, 1999[115], Wilkinson, 1998[116])	UK	6	Yes	Venous leg ulcers	NR	Patients with venous or arterial leg ulcers for at least 4 weeks	Diabetes, venous insufficiency	Dermatology ward, surgical department, surgical out-patient	13-43 weeks	4–60.1weeks
Zarchi, 2012[72]	Denmark	7	Yes	Venous leg ulcers	NR	NR	Diabetes, venous insufficiency, surgical	NR	NR	10 days - 12 months
Aziz, 2011[73] (CR: Aziz, 2010[117])	Malaysia	3	Yes	Venous leg ulcers	All ages	NR	NR	NR	8-12 weeks	8-12 weeks
Cruciani, 2011[24]	Italy	5	Yes	Diabetic foot/leg ulcers, mixed arterial/venous leg ulcers	30-86	Type I & II diabetics	Osteomyelitis, cellulitis	Hospital	1-21 days	5 days-26 weeks
Cullum, 2011[25]	UK	8	Yes	Venous leg ulcers	NR	NR	Elderly	Hospital	Daily-12 weeks	3-12 weeks
Damiani, 2011[26]	Italy	6	No	Infected surgical wounds	NR	Post-sternotomy osteomyelitis	NR	Hospital	NR	NR
Dumville, 2011a[27]	UK	6	Yes	Diabetic foot/leg ulcers	18-80	Type I & II diabetics	NR	NR	Daily-16 weeks	< 16 weeks
Dumville, 2011b[28]	UK	5	Yes	Diabetic foot/leg ulcers	NR	Type I & II diabetics	NR	NR	0.5 days-20 weeks	10 days-16 weeks
Hu, 2011[29]	China	31	Yes	Venous leg ulcers	28-70	Adults with a variety of wounds to pressure ulcers, venous	NR	NR	Daily-5 weeks	NR

						ulcers, diabetic ulcers and other disease related ulcers				
Hunt, 2011[79] (CR: Hunt, 2009[120])	Canada	36	Yes	Diabetic foot/leg ulcers	NR	Type I & II diabetics	NR	NR	NR	NR
Jull, 2011[30] (CR: Jull, 2007[118])	New Zealand	12	Yes	Venous leg ulcers	Any age	NR	NR	Any care setting	7 days-26 weeks	NR
Lima, 2011[74]	Brazil	8	No	Diabetic foot/leg ulcers	NR	Type I & II diabetics	NR	NR	NR	NR
McGinnis, 2011[75]	UK	1	Yes	Pressure ulcers	Average 84		Dementia, neurological disabilities, cardiovascular disease, malignancy or orthopaedic problems	Hospital	78 weeks	NR
McInnes, 2011[76]	Australia	18	Yes	Pressure ulcers	Adults	Inpatient	NSD	Nursing homes, care of the elderly, medical or surgical wards.	NR	NR
Nelson, 2011a[77] (CR: Nelson, 2008[119])	UK	101	Yes	Venous leg ulcers	NR	NR	NR	NR	NR	NR
Nelson, 2011b[31]	UK	7	Yes	Venous leg ulcers	NR	NR	NR	Community/home care setting as well as outpatient and inpatient settings	2028 hours /week	13-26 weeks
Reddy, 2011[78]	USA	15	Yes	Pressure ulcers	>18 or not discussed	NR	NR	NR	NR	NR
Suissa, 2011[32]	Canada	10	Yes	Mixed chronic wounds	NR	NR	NR	NR	NR	2-6 weeks

Voigt, 2011[33]	USA	8	Yes	Diabetic foot/leg ulcers	NR	NR	Diabetes, Neuropathic insufficiency, venous insufficiency, arterial occlusive disease	NR	NR	5-24 weeks
Carter, 2010[34]	USA	10	Yes	Mixed arterial/venous leg ulcers, venous leg ulcers	NR	NR	NR	NR	NR	NR
Chen, 2010[35]	China	6	Yes	Diabetic foot/leg ulcers, mixed arterial/venous leg ulcers	NR	Type I & II diabetics	NR	NR	3 to 24 weeks	NR
O'Meara, 2010[36] (CR: O'Meara, 2009[121])	UK	25	Yes	Venous leg ulcers	NR	NR	NR	Any setting: inpatient, outpatient, nursing home, etc.	10 days-24 weeks	10 days-24 weeks
Pan, 2010[37]	Italy	10	No	Infected surgical wounds	≥13	NR	NR	Hospital	2 days-1 week	NR
Vermeulen, 2010[80]	Netherlands	3	Yes	Mixed arterial/venous leg ulcers	> 18	NR	NR	All settings	NR	4 weeks
Villela, 2010[38]	Brazil	18	Yes	Diabetic foot/leg ulcers	NR	Type I & II diabetics	NR	NR	NR	NR
Xie, 2010[81]	Canada	10	Yes	Diabetic foot/leg ulcers	37-74	NR	Diabetics, venous insufficiency, surgical	NR	NR	3-28 weeks
Amsler, 2009[39]	Switzerland	8	Yes	Venous leg ulcers	Mean overall: 60.7	NR	NR	Single and multi-centers, NR	NR	12-78 weeks
Jull, 2009[40]	New Zealand	2	Yes	Venous leg ulcers	Any age	NR	NR	Community setting, outpatient	<12 weeks	NR

								clinics, hospitals		
Lo, 2009[41]	Taiwan	8	Yes	Mixed chronic wounds	58.9-74.9	NR	NR	Western healthcare environments.	4-8 weeks	NR
Martinez-Zapata, 2009[42] (CR to Martinez-Zapata, 2012[21])	Spain	20	Yes	Mixed chronic wounds	Adults	NR	NR	NR	NR	NR
McGaughey, 2009[82]	Ireland	11	Yes	Mixed arterial/venous leg ulcers	NR	NR	NR	NR	NR	NR
Ministry of Health and Long-term Care, 2009a[43]	Canada	108	Yes	Pressure ulcers	47-88	Immobile, bedridden,	NR	Any care setting	<78.2 weeks	10 months
Ministry of Health and Long-term Care, 2009b[83]	Canada	2	Yes	Mixed arterial/venous leg ulcers	Mean age between 73-83	Persons newly referred to homecare for leg ulcer management; persons with leg or pressure wounds	NR	Home care	NR	13-26 weeks or until wounds healed
Nelson, 2009[84] (CR: Nelson, 2006[122])	UK	1	Yes	Mixed arterial/venous leg ulcers	NR	NR	NR	NR	NR	NR
O'Meara, 2009a[44]	UK	7	Yes	Venous leg ulcers	Mean overall (SD): 69.9(13.5)	NR	Presence of: slough, granulation, epithelialising tissue; history of deep vein thrombosis	NR	NR	13-52 weeks

O'Meara, 2009b[45]	UK	39	Yes	Mixed arterial/venous leg ulcers, venous leg ulcers	18-97	NR	NR	NR	NR	NR
Ramundo, 2009[85] (CR: Ramundo, 2008[123])	USA	12	Yes	Pressure ulcers	Adults (8 studies), elderly (1 study)	NR	NR	NR	NR	NR
Roukis, 2009[86]	USA	2	No	Diabetic foot/leg ulcers	37-81	Type I & II diabetics	NR	Hospital (surgical)	NR	12-256 weeks
Barber, 2008[46]	Australia	24	Yes	Diabetic foot/leg ulcers, venous leg ulcers	NR	NR	NR	NR	NR	6-26 weeks
Blozik, 2008[47]	Germany	5	Yes	Diabetic foot/leg ulcers	Any age	Type I & II diabetics	NR	Any setting	NR	NR
Flemming, 2008[48]	UK	4	Yes	Venous leg ulcers	Any age	Patients described as having (chronic) venous leg ulcers ("resistant to conventional therapy")	NR	Any care setting	10 minutes to 1x/week for 39 weeks	NR
Heyneman, 2008[87]	Belgium	29	Yes	Pressure ulcers	NR	NR	NR	Inpatient hospitals, home care services, nursing homes, wound clinics, rehabilitation clinics	4.2-16 weeks	Median study length 8 weeks
Hinchliffe, 2008[88]	UK	60	Yes	Diabetic foot/leg ulcers	≥18	Type I & II diabetics	NR	NR	Daily-12 weeks	4-26 weeks
Howard, 2008[89]	UK	54	Yes	Venous leg ulcers	NR	NR	NR	NR	NR	28.3-208.7 weeks (only for RCTs)

Langer, 2008[90]	Germany	4	Yes	Pressure ulcers	NR	NR	NR	Institution, hospital, nursing home	4 -12 weeks	NR
Lo, 2008[91]	Taiwan	14	Yes	Venous leg ulcers	18-99	NR	NR	NR	9 days-8 weeks	NR
Moore, 2008[92]	Ireland	3	Yes	Pressure ulcers	All ages	NR	NR	NR	NR	NR
Noble-Bell, 2008[93]	UK	4	Yes	Diabetic foot/leg ulcers	NR	Type I & II diabetics	NR	NR	2-16 weeks	NR
Reddy, 2008[94]	USA	103	Yes	Pressure ulcers	>18	NR	NR	Mixed settings, long term care, rehab, ambulatory, home care, palliative, acute care	1-104 weeks	NR
Sadat, 2008[49]	UK	3	Yes	Mixed arterial/venous leg ulcers	NR	Type I & II diabetics, vascular lower limb wounds	Diabetes	NR	NR	NR
Ubbink, 2008a[95]	Netherlands	7	Yes	Diabetic foot/leg ulcers	~40-60	NR	Diabetic, venous insufficiency,	All settings	NR	2-52 weeks
Ubbink, 2008b[96]	Netherlands	13	Yes	Infected surgical wounds	NR	NR	Diabetics	All settings	NR	NR
Van den Boogaard, 2008[97]	Netherlands	5	Yes	Pressure ulcers	NR	NR	NR	NR	NR	4-6 weeks
Chambers, 2007[50]	UK	9	Yes	Mixed arterial/venous leg ulcers	NR	NR	NR	NR	NR	4 to 12 weeks
Jones, 2007[51]	UK	9	Yes	Venous leg ulcers	Any age	Leg ulcers	NR		NR	20 days-52 weeks
Palfreyman, 2007[52]	UK	42	Yes	Venous leg ulcers	NR	NR	NR	NR	4-40 weeks	NR
Nelson, 2006[98] (CR: O'Meara,	UK	23	Yes	Diabetic foot/leg ulcers	NSD	Type I & II diabetics	NR	Inpatients and outpatients.	NR	NR

2000[124])										
O'Donnell, 2006[53]	USA	20	Yes	Venous leg ulcers	50-80	NR	NR	NR	NR	NR
Sari, 2006[54]	Iran	3	Yes	Pressure ulcers	All ages	NR	NR	Nursing home in one study	3-5 times/week	Unclear
Bouza, 2005a[99]	Spain	21	Yes	Pressure ulcers	NR	NR	NR	NR	NR	NR
Bouza, 2005b[55]	Spain	31	Yes	Mixed chronic wounds, mixed arterial/venous leg ulcers, venous leg ulcers	NR	Ambulatory patients	NR	NR	6 weeks-160.9 months	NR
Coleridge-Smith, 2005[56]	UK	5	Yes	Mixed arterial/venous leg ulcers, venous leg ulcers	Adults	Chronic venous disease	Post-thrombotic syndrome, varicose veins	NR	NR	2.5-26 weeks
Cruciani, 2005[57]	Italy	5	Yes	Mixed arterial/venous leg ulcers, diabetic foot/leg ulcers	NR	Type I & II diabetics	NR	NR	3-21 days	NR
Ho, 2005[58]	Canada	23	Yes	Diabetic foot/leg ulcers, mixed chronic wounds, venous leg ulcers	51-88	Older adults with foot ulcers that are diabetic, venous or both in origin	NR	Hospital	6-24 weeks	NR
Roeckl-Wiedmann, 2005[59]	Germany	6	Yes	Diabetic foot/leg ulcers	NR	Type I & II diabetics	NR	NR	NR	NR
Schuren, 2005[100]	Canada	9	Yes	Venous leg ulcers	NR	NR	NR	NR	NR	NR
Stratton, 2005[16]	UK	15	Yes	Pressure ulcers	>18 (40-90)	NR	Diabetes, dementia,	Hospital and community	2-74 weeks	NR
Cullum, 2004[60]	UK	8	Yes	Pressure ulcers	16-98	Impaired mobility, elderly, surgical	Fixed joints, peripheral vascular disease,	Hospital, long term care facility, nursing home	NR	13 days-36 weeks

						patients, nursing home/long term care resident	neurological disorder			
Mwipatayi, 2004[101]	Australia	2	No	Mixed arterial/venous leg ulcers	NR	NR	NR	NR	NR	NR
Singh, 2004[61]	India	12	Yes	Mixed chronic wounds	>24	NR	Diabetes	Hospital	NR	NR
TenBrook, 2004[102]	USA	20	Yes	Venous leg ulcers	44-67	Venous Insufficiency	NR	Hospital	NR	32-576 weeks
Berliner, 2003[103]	USA	8	Yes	Venous leg ulcers	NR	NR	NR	NR	NR	NR
Pham, 2003[104]	Australia	17	Yes	Pressure ulcers	NR	Diabetic, surgical	NR	NR	NR	2 days-40 weeks
Wang, 2003[105]	USA	57	Yes	Venous leg ulcers	NR	NR	Diabetes, arterial insufficiencies	NR	NR	NR
Cullum, 2001a[62] (CR: Cullum, 2008[125])	UK	69	Yes	Pressure ulcers	NR	NR	NR	Hospital/nursing home	2-36 weeks	NR
Cullum, 2001b[62] (CR: Cullum, 2008[125])	UK	45	Yes	Venous leg ulcers	NR	NR	NR	Any care setting (mainly hospitals, long term care facilities	NR	NR
Cullum, 2001c[62] (CR: Cullum, 2008[125])	UK	24	Yes	Mixed chronic wounds	NR	NR	NR	Any care setting	NR	NR
Emergency Care Research Institute (ECRI), 2001[106]	USA	10	Yes	Pressure ulcers	NR	NR	NR	Home and "other settings"	36 weeks	NR

Evans, 2001[107]	UK	2	Yes	Venous leg ulcers	18-75	Diabetics, pressure, trauma	NR	NR	3- 6 weeks	NR
Lewis, 2001[108]	UK	17	Yes	Infected surgical wounds	NR	NR	NR	Hospital and clinic	NR	NR
Moore, 2001[109]	Ireland	1	Yes	Infected surgical wounds	NR	NR	NR	NR	NR	NR
O'Meara, 2001[110] (CR: O'Meara, 2000[124])	UK	22	Yes	Mixed arterial/venous leg ulcers	NR	Venous and arterial insufficiency, limited mobility, diabetic	NR	NR	NR	NR
Lucas, 2000[63]	Netherlands	4	Yes	Mixed chronic wounds	NR	NR	NR	NR	NR	NR
Bradley, 1999a[111]	UK	35	Yes	Mixed chronic wounds, venous leg ulcers	NR	NR	NR	NR	NR	NR
Bradley, 1999b[64]	UK	93	Yes	Pressure ulcers, venous leg ulcers	NR	NR	NR	NR	NR	NR
Mason, 1999[112]	UK	29	Yes	Diabetic foot /leg ulcers	NR	Type I & II diabetics	NR	NR	NR	NR
Johannsen, 1998[65]	Denmark	6	Yes	Mixed arterial/venous leg ulcers, venous leg ulcers	61-73	Adults with chronic venous leg ulceration	NR	NR	4-12 weeks	NR
Palfreyman, 1998[66]	UK	8	Yes	Venous leg ulcers	NR	NR	NR	Includes ambulatory, clinic, outpatient	13-78.3 weeks (if reported)	26 weeks (if reported)
Fletcher, 1997[67]	UK	24	Yes	Venous leg ulcers	NR	NR	NR	Hospital	NR	NR
Note: NA = not applicable, NR = not reported, RCT = randomized clinical trial.										
