# Supplementary material for: Seeking effective interventions to treat complex wounds: an overview of systematic reviews
Source: BMC Med. 2015 Apr 22;13:89. doi: 10.1186/s12916-015-0288-5 (PMC4406332; doi:10.1186/s12916-015-0288-5)
Supplement: Additional file 4: — Meta-analysis results. Provides relevant findings from the included reviews with a meta-analysis. [file 12916_2015_288_MOESM4_ESM.pdf]

Meta-analysis results										
**Intervention**	**Review**	**# of studies in MA**	**Comparator**	**Results of meta-analysis**						**Conclusion**
				**RR**	**95% CI**	**OR**	**95%CI**	**MD**	**95% CI**	
Venous leg ulcers – wound area/size reduction (2 MAs)										
Cadexomer iodine (topical)	O'Meara, 2010[36] (CR: O'Meara, 2008[121])	2	Standard care	0.47	0.26, 0.69	NR	NR	NR	NR	Cadexomer iodine more effective than standard care*
Micronized purified flavonoid fraction (MPFF) (oral)	Coleridge-Smith, 2005[56]	5	Placebo or standard compression	54% and 44% (7%, 94%)	0%, 137% and 7%, 94%	NR	NR	NR	NR	Micronized purified flavonoid more effective than placebo or standard compression
Venous leg ulcers – time to healing or rate of healing (5 MAs)										
4-layer bandage	O'Meara, 2009a[44]	5	Short stretch bandage	1.31	1.09, 1.58	NR	NR	NR	NR	4-layer bandage more effective than short stretch bandage*
	O'Meara, 2009b[45]	2	Compression system with paste bandage as the base	0.52	0.06, 0.97	NR	NR	NR	NR	4-layer bandage more effective than compression system*
Micronized purified flavonoid fraction (MPFF) (oral)	Coleridge-Smith, 2005[56]	NR	Placebo or standard compression	32%	3%, 70%	NR	NR	NR	NR	Micronized purified flavonoid more effective than placebo or standard compression

Stockings	Amsler, 2009[39]	7	Bandages	NR	NR	NR	NR	Standardized MD -0.33	-0.50, -0.16	Bandages more effective than stockings
	Amsler, 2009[39]	3	Bandages	NR	NR	NR	NR	Standardized MD -0.56	-0.83, -0.28	Stockings more effective than bandages
Silver-impregnated dressings	Carter, 2010[34]	7	Non-silver dressings	NR	NR	NR	NR	0.02	-0.01, 0.06	Nsd
<u>Venous leg ulcers – ulcer healing (8 MAs)</u>										
Elastic high compression bandages	Cullum, 2001b[62] (CR: Cullum, 2008[125])	3	Multi-layer inelastic compression bandages	1.54	1.19, 1.99	NR	NR	NR	NR	Elastic high compression more effective than multi-layer inelastic compression*
Cryopreserved allografts (Skin grafting)	Jones, 2007[51]	2	Dressings	1.62	0.79, 3.33	NR	NR	NR	NR	Nsd
Cultured keratinocytes / epidermal grafts (Skin grafting)	Jones, 2007[51]	4	Dressings	1.73	0.91, 3.28	NR	NR	NR	NR	Nsd
Fresh allografts (Skin grafting)	Jones, 2007[51]	2	Dressings	2.06	0.54, 7.9	NR	NR	NR	NR	Nsd
Granulocyte-macrophage colony stimulating factor (topical)	Hu, 2011[29]	2	Placebo	NR	NR	6.5	2.15, 19.66	NR	NR	Granulocyte-macrophage colony stimulating factor more effective than placebo
Pentoxifylline (oral)	Jull, 2011[30] (CR: Jull, 2007[118])	11	All other treatments	1.7	1.30, 2.24	NR	NR	NR	NR	Pentoxifylline more effective than all other treatments*
Stockings	Amsler, 2009[39]	8	Bandages	NR	NR	0.44	0.32, 0.61	NR	NR	Stockings more effective than bandages
Tissue engineered skin	Jones, 2007[51]	2	Dressings	1.51	1.22, 1.88	NR	NR	NR	NR	Tissue engineered skin more effective than dressings

Venous leg ulcers – proportion of patients with healed wounds (40 MAs)										
4-layer bandage	Cullum, 2001b[62] (CR: Cullum, 2008[125])	3	Other multi-layer high compression bandages	1.02	0.87, 1.18	NR	NR	NR	NR	Nsd*
	O'Meara, 2009b[45]	2	Compression system with paste bandage as the base	1.34	0.78, 2.28	NR	NR	NR	NR	Nsd*
	O'Meara, 2009b[45]	4	Multi-layer short-stretch bandage	1.12	0.96, 1.31	NR	NR	NR	NR	Nsd*
Any laser (unspecified low level laser, ultraviolet therapy, non-coherent unpolarized red light)	Flemming, 2008[48]	2	Sham	1.21	0.73, 2.03	NR	NR	NR	NR	Nsd
Artificial skin graft and standard wound care	Ho, 2005[58]	3	Standard wound care	1.6	0.57, 4.46	NR	NR	NR	NR	Nsd
Autologous platelet-rich plasma (topical)	Martinez-Zapata, 2012[21]	2	Standard treatment with/without placebo	1.02	0.81, 1.27	NR	NR	NR	NR	Nsd
Cadexomer iodine (topical) plus compression therapy	O'Meara, 2010[36] (CR: O'Meara 2009[121])	2	Standard care plus compression therapy	6.72	1.56, 28.95	NR	NR	NR	NR	Cadexomer iodine more effective than standard care*
Systemic ciprofloxacin (oral)	O'Meara, 2010[36] (CR: O'Meara 2009[121])	2	Standard care/placebo	1.72	0.57, 5.16	NR	NR	NR	NR	Nsd*
Skin replacement therapy (Dermagraft)	Barber, 2008[46]	2	Standard compression therapy	NR	NR	4.48	1.01, 19.80	NR	NR	Skin replacement therapy more effective than standard

										compression therapy
Elastic high compression bandages	Fletcher, 1997[67]	3	Inelastic compression bandages	NR	NR	2.26	1.4, 3.7	NR	NR	Elastic multi-layer high compression bandage more effective than inelastic compression*
	O'Meara, 2009b[45]	2	Components including inelastic bandage	1.83	1.26, 2.67	NR	NR	NR	NR	Elastic bandage more effective than inelastic bandage*
Foam dressing	Palfreyman, 2007[52]	2	Low adherent dressings	1.35	0.93, 1.94	NR	NR	NR	NR	Nsd*
	Palfreyman, 2007[52]	2	Foam dressing	1.2	0.77, 1.87	NR	NR	NR	NR	Nsd*
Granulocyte-macrophage colony stimulating factor (peri-lesional injection)	O'Donnell, 2006[53]	8	Control (non-adherent, visco paste, gauze pad, hydrocolloid)	0.2	0.1, 0.3	NR	NR	NR	NR	Granulocyte-macrophage colony stimulating factor more effective than control
High frequency ultrasound	Cullum, 2011[25]	6	No ultrasound	1.34	0.99, 1.80	NR	NR	NR	NR	Nsd*
Honey (topical)	Jull, 2009[40]	2	Control (non-specified)	1.15	0.96, 1.38	NR	NR	NR	NR	Nsd*
Hydracolloid dressings	Bouza, 2005b[55]	8	Traditional dressings	0.99	0.85, 1.15	NA	NA	NA	NA	Nsd*
	Bradley, 1999b[64]	8	Traditional dressings	NR	NR	1.45	0.83, 2.54	NR	NR	Nsd*
	Bradley, 1999b[64]	2	Foam dressing	NR	NR	1	0.48, 2.08	NR	NR	Nsd*
	Palfreyman, 2007[52]	8	Low adherent dressings	1.02	0.83, 1.25	NR	NR	NR	NR	Nsd*
	Palfreyman, 2007[52]	4	Foam dressing	0.98	0.79, 1.22	NR	NR	NR	NR	Nsd*
	Palfreyman,	3	Alginate dressing	0.72	0.15,	NR	NR	NR	NR	Nsd*

	2007[52]				3.42					
	Palfreyman, 2007[52]	3	Hydrocolloid dressing	1.56	0.67, 3.63	NR	NR	NR	NR	Nsd*
Hydrogel dressing	Palfreyman, 2007[52]	2	Low adherent dressings	1.53	0.96, 2.42	NR	NR	NR	NR	Nsd*
Intermittent pnuematic compression stockings	Fletcher, 1997[67]	2	Compression stockings or Unna's boot	NR	NR	10	3.33, 33.8	NR	NR	Intermittent pneumatic compression more effective than compression stockings or Unna's boot*
	Palfreyman, 1998[66]	2	No intermittent compression	NR	NR	8.45	NR	NR	NR	Nsd, CI NR
	Nelson, 2011b[31]	3	Compression stockings	1.09	0.91, 1.30	NR	NR	NR	NR	Nsd*
Low frequency ultrasound	Cullum, 2011[25]	2	No ultrasound	3.91	0.47, 32.85	NR	NR	NR	NR	Nsd*
Multi-layer high compression bandages	Cullum, 2001b[62] (CR: Cullum, 2008[125])	5	Inelastic compression bandages	1.08	0.79, 1.49	NR	NR	NR	NR	Nsd*
	Cullum, 2001b[62] (CR: Cullum, 2008[125])	4	Single-layer compression bandages	1.41	1.12, 1.77	NR	NR	NR	NR	Multi-layer high compression more effective than single-layer compression*
	Fletcher, 1997[67]	4	Single layer systems	NR	NR	2.2	1.3, 3.5	NR	NR	Elastic multi-layer high compression bandage more effective than single layer systems*
Pentoxifylline (oral) with and without	Jull, 2011[30]	7	Placebo	1.56	1.14, 2.13	NR	NR	NR	NR	Pentoxifylline with

compression	(CR: Jull, 2007[118])									compression more effective than placebo*
	Jull, 2011[30] (CR: Jull, 2007[118])	4	Placebo	2.25	1.49-3.39	NR	NR	NR	NR	Pentoxifylline without compression more effective than placebo*
Stockings	Cullum, 2001b[62] (CR: Cullum, 2008[125])	2	Compression bandaging	1.39	1.00, 1.92	NR	NR	NR	NR	High compression stockings more effective than compression bandage*
Two-component (outer elastic) bandages	O'Meara, 2009b[45]	2	Two-component (outer elastic)	1.23	0.67, 2.25	NR	NR	NR	NR	Nsd*
	O'Meara, 2009b[45]	2	Short-stretch bandage	1.72	1.14, 2.58	NR	NR	NR	NR	2-layer stocking more effective than short-stretch bandage*
Ultrasound	Johannsen, 1998[65]	5	No ultrasound	NR	NR	NR	NR	15	1, 30	Ultrasound more effective than no ultrasound
Unna's boot	Palfreyman, 1998[66]	3	Other therapies	NR	NR	5.8	NR	NR	NR	Nsd, CI NR
Zinc (oral)	Wilkinson, 2012 [23] (CR: Wilkinson, 1999[115], Wilkinson, 1998[116])	4	Placebo	1.22	0.88, 1.68	NR	NR	NR	NR	Nsd*
Mixed arterial/venous leg ulcers – wound area/size reduction (3 MAs)										
Silver treatments (topical) and silver-impregnated dressings	Carter, 2010[34]	5	Placebo, conservative wound care or treatment, not another type of silver treatment	NR	NR	NR	NR	10.29	3.86, 16.71	Silver treatments more effective than placebo or conservative

										wound care or non-silver therapies
	Carter, 2010[34]	3	Non-silver dressings	NR	NR	NR	NR	0.01	-0.02, 0.05	Nsd
Ultrasound	Johannsen, 1998[65]	6	Standard treatment, placebo	NR	NR	NR	NR	14.5	6.6, 22.3	Ultrasound more effective than standard treatment or placebo
<u>Mixed arterial/venous leg ulcers – time to healing or rate of healing (5 MAs)</u>										
Topical negative pressure	Sadat, 2008[49]	2	Conventional therapy	NR	NR	1.93	1.05, 3.56	-1.04	1.83, -0.25	Topical negative pressure more effective than conventional therapy
4-layer bandage	O'Meara, 2009b[45]	2	Compression system with paste bandage as the base	0.52	0.06, 0.97	NR	NR	NR	NR	4-layer bandage more effective than compression system[*]
Micronized purified flavonoid fraction (oral)	Coleridge-Smith, 2005[56]	4	Placebo or standard compression	Reduction of RR % 32	3.0, 70.0	NR	NR	NR	NR	Micronized purified flavonoid more effective than placebo or standard compression
Polyurethane (dressing)	Bouza, 2005b[55]	3	Conventional care	0.92	0.14, 1.98	NA	NA	NA	NA	Nsd[*]
Alginate (beads, paste + dressing, alginate dressing)	Bouza, 2005b[55]	2	Conventional care	1.10	0.86, 1.43	NA	NA	NA	NA	Nsd[*]
<u>Mixed arterial/venous leg ulcers – proportion of patients with healed wounds (3 MAs)</u>										
Silver dressings (topical or impregnated)	Chambers, 2007[50]	2	Placebo/no treatment	1.79	0.19, 17.11	NR	NR	NR	NR	Nsd
	Chambers, 2007[50]	2	Placebo/no treatment	1.66	0.68, 4.05	NR	NR	NR	NR	Nsd
Topical negative	Sadat,	2	Conventional	NR	NR	1.93	1.05,	NR	NR	Topical

pressure	2008[49]		therapy				3.56			negative more effective than conventional therapy
<u>Diabetic foot/leg ulcers – ulcer healing (4 MAs)</u>										
Chinese herbal medicine + standard therapy (*Bu-yang-huan-wu* decoction, *Tao-hong-si-wu* decoction, *Si-miao-yong-an* decoction, Radix astragali, Rhizoma atractylodis, marcocephalae, Radix stephaniae tetrandrae, Radix Polygoni multifori, Radix Rehmanniae, Radix smilax china, Frusctus corni, Rhizoma dioscoreae, Cortex Moutan, Rhizoma alismatis, Rhizoma smilacis glabrae, Frutus schisandrae, Herba Siegesbeckiae, Draconis Sanguis, Lumbricus, Radix Ligustici, Chuanxiogm, Ramulus Cinnamomi Cassiae, *She-xiang-huo-xue capsule*)	Chen, 2010[35]	6	Standard therapy	0.34	0.21, 0.53	NR	NR	NR	NR	Chinese herbal medicine more effective than standard therapy
Granolocyte colony-stimulating factor (SC, IV) + antibiotics (oral, IV)	Cruciani, 2005[57]	NR	Placebo/usual care	NR	NR	NR	NR	NR	NR	Nsd, data not shown
	Cruciani, 2011[24]	4	Control	1.4	1.06, 1.85	NR	NR	NR	NR	Granolocyte colony-stimulating factor more effective than

										control*
	Cruciani, 2011[24]	2	Usual care, placebo	9.45	0.54, 164.49	NR	NR	NR	NR	Nsd*
Diabetic foot/leg ulcers – no healing improvement/non-healed wounds (5 MAs)										
Chinese herbal medicine (*Bu-yang-huan-wu* decoction, *Tao-hong-si-wu* decoction, *Si-miao-yong-an* decoction, Radix astragali, Rhizoma atractylodis, marcocephalae, Radix stephaniae tetrandrae, Radix Polygoni multifori, Radix Rehmanniae, Radix smilax china, Frusctus corni, Rhizoma dioscoreae, Cortex Moutan, Rhizoma alismatis, Rhizoma smilacis glabrae, Frutus schisandrae, Herba Siegesbeckiae, Draconis Sanguis, Lumbricus, Radix Ligustici, Chuanxiogm, Ramulus Cinnamomi Cassiae, *She-xiang-huo-xue capsule*)	Chen, 2010[35]	6	Standard therapy	0.34	0.21, 0.53	NR	NR	NR	NR	Chinese herbal medicine more effective than standard therapy
Hyaluronic acid based scaffold and keratinocytes	Voigt, 2012[22]	2	Standard of care	0.9	0.76, 1.07	NR	NR	NR	NR	Nsd*
Hyaluronic acid based derivative	Voigt, 2012[22]	2	Standard of care	0.24	0.12, 0.49	NR	NR	NR	NR	Hyaluronic acid based derivatives more effective than standard

										care*
Low frequency low intensity noncontact ultrasound	Voigt, 2011[33]	2	Sharps debridement	0.74	0.58, 0.95	NR	NR	NR	NR	Low frequency low intensity noncontact ultrasound more effective than sharps debridement*
	Voigt, 2011[33]	2	Sharps debridement	0.64	0.46, 0.89	NR	NR	NR	NR	Low frequency low intensity noncontact ultrasound more effective than sharps debridement*
Diabetic foot/leg ulcers – proportion of patients with healed wounds (18 MAs)										
Alginate dressing	Dumville, 2012b[18]	2	Basic wound contact dressing	1.09	0.66, 1.80	NA	NA	NA	NA	Nsd*
	Dumville, 2012b[18]	2	Foam dressing	0.67	0.41, 1.08	NA	NA	NA	NA	Nsd*
Artificial skin graft and standard care	Ho, 2005[58]	9	Standard care	1.4	1.21, 1.63	NR	NR	NR	NR	Artificial skin graft with standard care more effective than usual care alone
Chinese herbal medicine plus standard treatment (*Tao-hong-si-wu* decoction, *Si-miao-yong-an* decoction, Radix astragali, Radix Rehmanniae, Herba Siegesbeckiae, Draconis Sanguis, Lumbricus, Radix Ligustici, Chuanxiogm, Ramulus Cinnamomi Cassiae, *She-xiang-huo-xue capsule*)	Chen, 2010[35]	4	Standard therapy	0.62	0.39, 0.97	NR	NR	NR	NR	Chinese herbal medicine more effective than standard therapy

Skin graft (Dermagraft)	Barber, 2008[46]	3	Standard saline dressings	NR	NR	1.78	0.92, 3.45	NR	NR	Nsd
Fibrous-hydrocolloid (hydrofibre) dressing	Dumville, 2012a[17]	2	Basic wound contact dressing	1.01	0.74, 1.38	NR	NR	NR	NR	Nsd*
Foam dressing	Dumville, 2011a[27]	2	Basic wound contact dressing,	2.03	0.91, 4.55	NR	NR	NR	NR	Nsd*
	Dumville, 2011a[27]	2	Alginate dressing,	1.5	0.92, 2.44	NR	NR	NR	NR	Nsd*
Granolocyte colony-stimulating factor (SC, IV) + antibiotics (oral, IV)	Cruciani, 2005[57]	NR	Placebo/usual care	NR	NR	NR	NR	NR	NR	Nsd, data not shown
	Cruciani, 2011[24]	2	Usual care/placebo	2.75	1.05, 7.20	NR	NR	NR	NR	Granolocyte colony-stimulating factor more effective than usual care*
Hyaluronic acid based scaffold and keratinocytes	Voigt, 2012[22]	2	Standard of care	0.9	0.76, 1.07	NR	NR	NR	NR	Nsd*
Hydrogel dressing	Dumville, 2011b[28]	3	Basic wound dressing	1.8	1,27, 2.56	NR	NR	NR	NR	Hydrogel dressing more effective than basic wound dressing*
	Dumville, 2012a[17]	3	Basic wound contact dressing	1.8	1.27, 2.56	NR	NR	NR	NR	Hydrogel dressing more effective than basic wound dressing*
	Edwards, 2012[19]	3	Gauze or good wound care	1.84	1.30, 2.61	NR	NR	NR	NR	Hydrogel more effective than gauze with usual care
Hyperbaric oxygen therapy	Kranke, 2012[20]	3	Hyperbaric air, sham	9.53	0.44, 207.76	NR	NR	NR	NR	Nsd*
	Roeckl-Wiedmann, 2005[59]	2	Control	4.78	0.94, 24.24	NR	NR	NR	NR	Nsd*
Platelet-rich plasma	Villela, 2010[38]	4	Control	NR	NR	7.70	2.94, 20.31	NR	NR	Platelet-rich plasma more

										effective than control
Skin replacement therapy (keratinocyte allograft, meshed skin autograft, split thickness autograft)	Blozik, 2008[47]	5	Standard care	NR	NR	1.46	1.21, 1.76	NR	NR	Skin replacement therapy more effective than standard care
<u>Pressure ulcers – ulcer healing (8 MAs)</u>										
Air-fluidised bed/supports	Cullum, 2004[60]	2	Standard care	1.4	1.04, 1.88	NR	NR	NR	NR	Air-fluidised supports more effective than standard care*
	Cullum, 2001a[62] (CR: Cullum, 2008[125])	3	Standard care	NR	NR	NR	NR	NR	NR	Air-fluidised supports more effective than standard care (data NR)
	Ministry of Health and Long-term Care, 2009a[43]	2	Conventional mattresses	1.4	1.04, 1.88	NR	NR	NR	NR	Air-fluidised beds more effective than conventional beds
Alternative foam mattress	Cullum, 2004[60]	5	Standard foam mattress	0.4	0.21, 0.74	NR	NR	NR	NR	Alternative foam more effective than standard foam mattress*
Electrotherapy	Cullum, 2001a[62] (CR: Cullum, 2008[125])	2	Sham electrotherapy	7.92	2.39, 26.31	NR	NR	NR	NR	Electrotherapy more effective than sham*
High protein, oral nutritional support, enteral tube feeding	Stratton, 2005[16]	4	Routine care	NR	NR	0.75	0.62, 0.89	NR	NR	Nsd*
Hydrocolloid dressings	Bradley, 1999b[64]	5	Traditional treatments	NR	NR	2.57	1.58, 4.18	NR	NR	Hydrocolloid more effective than traditional

										treatments*
Polyurethane dressings	Bradley, 1999b[64]	4	Dressings	NR	NR	0.8	0.44, 1.44	NR	NR	Nsd‡
Pressure ulcers – proportion of patients with healed wounds (25 MAs)										
Collagenase debridement (topical)	Ministry of Health and Long-term Care, 2009a[43]	2	Hydrocolloid occlusive dressing	1.33	0.80, 2.23	NR	NR	NR	NR	Nsd
Dextranomer (beads + dry dressing)	Ministry of Health and Long-term Care, 2009a[43]	2	Hydrogel	0.88	0.51, 1.53	NR	NR	NR	NR	Nsd
Electrical stimulation	Ministry of Health and Long-term Care, 2009a[43]	3	Placebo	3.08	0.58, 16.41	NR	NR	NR	NR	Nsd
Electrotherapy	Ministry of Health and Long-term Care, 2009a[43]	3	Sham therapy	3.43	0.35, 33.61	NR	NR	NR	NR	Nsd
Growth Factors (topical)	Ministry of Health and Long-term Care, 2009a[43]	4	Placebo	0.29	0.52, 9.98	NR	NR	NR	NR	Nsd
	Ministry of Health and Long-term Care, 2009a[43]	2	Placebo at 4 weeks	4.43	0.48, 40.56	NR	NR	NR	NR	Nsd
	Ministry of Health and Long-term Care,	2	Placebo at 4 weeks	2.17	0.06, 81.31	NR	NR	NR	NR	Nsd

	2009a[43]									
	Ministry of Health and Long-term Care, 2009a[43]	4	Placebo	2.29	0.52, 9.98	NR	NR	NR	NR	Nsd
Hydrocolloid dressings	Ministry of Health and Long-term Care, 2009a[43]	4	Traditional dressing	3.84	2.30, 6.41	NR	NR	NR	NR	Hydrocolloid more effective than traditional dressing
	Ministry of Health and Long-term Care, 2009a[43]	2	Hydrocolloid dressing	1.38	0.78, 2.45	NR	NR	NR	NR	Nsd
	Ministry of Health and Long-term Care, 2009a[43]	2	Povidine-soaked Gauze	0.99	0.71, 1.37	NR	NR	NR	NR	Nsd
Hydrogel (gel)	Ministry of Health and Long-term Care, 2009a[43]	2	Hydrocolloid	1.71	1.05, 2.79	NR	NR	NR	NR	Hydrogel more effective than hydrocolloid dressing
Hydropolymer dressing	Ministry of Health and Long-term Care, 2009a[43]	2	Hydrocolloid dressing	1.1	0.77, 1.59	NR	NR	NR	NR	Nsd
Low-air-loss beds	Cullum, 2001a[62] (CR: Cullum, 2008[125])	2	Foam overlay	1.25	0.84, 1.86	NR	NR	NR	NR	Nsd*
	Ministry of Health and Long-term Care,	2	Convoluted foam overlay	1.25	0.84, 1.86	NR	NR	NR	NR	Nsd

	2009a[43]									
Low level laser therapy	Ministry of Health and Long-term Care, 2009a[43]	3	Conventional therapy	1.26	0.92, 1.95	NR	NR	NR	NR	Nsd
	Ministry of Health and Long-term Care, 2009a[43]	3	Conventional Therapy or Sham	1.17	0.85, 1.63	NR	NR	NR	NR	Nsd
Alternating pressure mattress	Ministry of Health and Long-term Care, 2009a[43]	2	Another alternating pressure mattress replacement/overlay	0.69	0.18, 2.57	NR	NR	NR	NR	Nsd
	Ministry of Health and Long-term Care, 2009a[43]	4	Another alternating pressure system in Hospital Setting	1.4	1.08, 1.80	NR	NR	NR	NR	Alternating pressure mattress more effective than other alternating pressure mattresses in hospital
Noncontact normothermic dressing	Ministry of Health and Long-term Care, 2009a[43]	4	Standard care	1.31	0.86, 1.98	NR	NR	NR	NR	Nsd
Polyurethane dressings	Ministry of Health and Long-term Care, 2009a[43]	3	Hydrocolloid	1.18	0.85, 1.64	NR	NR	NR	NR	Nsd
Ultrasound	Cullum, 2001a[62] (CR: Cullum, 2008[125])	2	No ultrasound	0.97	0.65, 1.45	NR	NR	NR	NR	Nsd*

	Ministry of Health and Long-term Care, 2009a[43]	2	Sham therapy	0.97	0.65, 1.45	NR	NR	NR	NR	Nsd
	Sari, 2006[54]	2	Sham	0.97	0.65, 1.45	NR	NR	NR	NR	Nsd*
Zinc supplement (oral)	Ministry of Health and Long-term Care, 2009a[43]	2	Placebo	0.97	0.22, 4.29	NR	NR	NR	NR	Nsd
Mixed chronic wounds - wound area/size reduction (5 MAs)										
Autologous platelet-rich plasma/platelet-rich plasma (topical)	Martinez-Zapata, 2009[42] (CR to Martinez-Zapata, 2012[21])	3	Control	1.8	0.63, 5.15	NR	NR	NR	NR	Nsd*
	Martinez-Zapata, 2012[21]	3	Standard treatment with/without placebo	NR	NR	NR	NR	-1.94	-4.74, 0.86	Nsd
	Martinez-Zapata, 2009[42] (CR to Martinez-Zapata, 2012[21])	6	Standard treatment	1.4	0.58, 2.31	NR	NR	NR	NR	Nsd*
Silver releasing dressings	Lo, 2009[41]	8	Non-silver dressings	NR	NR	NR	NR	0.28	0.16, 0.39	Silver dressings more effective than non-silver dressings*
Topical negative pressure	Suissa, 2011[32]	9	Standard wound care	0.77	0.63, 0.96	NR	NR	NR	NR	Topical negative pressure more effective than standard

										wound care
Mixed chronic wounds – ulcer healing (1 MA)										
Laser therapy	Lucas, 2000[63]	3	Placebo or any other intervention	0.76	0.41, 1.40	NR	NR	NR	NR	Nsd
Mixed chronic wounds – proportion of patients with healed wounds (10 MAs)										
Skin replacement (skin substitute) and standard care	Ho, 2005[58]	5	Standard wound care	1.71	1.34, 2.17	NR	NR	NR	NR	Skin replacement more effective than standard care
Skin replacement (dermal substitute) and standard care	Ho, 2005[58]	6	Standard care	1.36	1.11, 1.66	NR	NR	NR	NR	Dermal substitute more effective than standard care
Artificial skin grafts and standard care	Ho, 2005[58]	13	Standard care	1.44	1.22, 1.71	NR	NR	NR	NR	Any artificial skin graft plus standard wound care more effective than standard care
Autologous platelet-rich plasma/platelet-rich plasma (topical)	Martinez-Zapata, 2012[21]	2	Standard treatment with/without placebo	1.21	0.60, 2.41	NR	NR	NR	NR	Nsd
	Martinez-Zapata, 2012[21]	4	Standard treatment	1.85	0.76, 4.51	NR	NR	NR	NR	Nsd
Hydrocolloid dressings	Bouza, 2005b[55]	6	Control: other modern dressings	1.13	0.86, 1.48	NR	NR	NR	NR	Nsd‡
	Singh, 2004[61]	12	Conventional dressing	NR	NR	1.73	1.08, 2.78	NR	NR	Hydrocolloid dressings more effective than conventional dressings
Laser therapy	Cullum, 2001c[62] (CR: Cullum, 2008[125])	2	Sham	1.21	0.73, 2.03	NR	NR	NR	NR	Nsd*
Ultrasound	Cullum,	4	No ultrasound	1.44	1.01,	NR	NR	NR	NR	Ultrasound

	2001c[62] (CR: Cullum, 2008[125])				2.05					more effective than no ultrasound*
	Cullum, 2001c[62] (CR: Cullum, 2008[125])	4	Sham- ultrasound therapy	1.18	0.89, 1.54	NR	NR	NR	NR	Nsd*
Infected surgical wounds – proportion of patients with healed wounds (1 MA)										
Topical negative pressure/vacuum-assisted closure	Pan, 2010[37]	6	Standard treatment	NR	NR	6.43	3.81, 10.85	NR	NR	Topical negative pressure more effective than standard treatment
Infected surgical wounds – length of hospitalization (1 MA)										
Vacuum-assisted closure	Damiani, 2011[26]	6	Conventional therapy	NR	NR	NR	NR	-7.18	-10.8, -3.5	Vacuum-assisted closure more effective than conventional therapy

Note: CI = confidence interval, MA = meta-analysis, MD = mean difference, NR = not reported, Nsd = not statistically different, OR = odds ratio, RR = relative risk, SC = subcutaneous.
*These are high quality systematic reviews (AMSTAR score ≥8).
