# Supplementary material for: Seeking effective interventions to treat complex wounds: an overview of systematic reviews
Source: BMC Med. 2015 Apr 22;13:89. doi: 10.1186/s12916-015-0288-5 (PMC4406332; doi:10.1186/s12916-015-0288-5)
Supplement: Additional file 5: — AMSTAR results for each systematic review. Results of the AMSTAR quality assessment for included reviews. [file 12916_2015_288_MOESM5_ESM.pdf]

AMSTAR results for each systematic review											
**Study**[†]	**A priori design**	**Duplicate selection/ DA**	**Literat-ure search**	**Publicati-on status**	**List of studie s**	**Study characteristic s**	**Quality assesse d**	**Qualit y used**	**Methods appropria-te**	**Publicat-ion bias assessed**	**Conflict s stated**	**AMSTA R rating**
Dat, 2012[68]	Y	Y	Y	Y	Y	Y	Y	Y	Y	N	Y	10
Dumville, 2011b[28]	Y	Y	Y	Y	Y	Y	Y	Y	Y	Y	Y	11
Dumville, 2012a[17]	Y	Y	Y	Y	Y	Y	Y	Y	Y	Y	Y	11
Edwards, 2012[19]	U	U	Y	Y	N	Y	Y	Y	Y	N	Y	7
Hinchliffe, 2012[69]	U	Y	N	N	N	Y	Y	Y	Y	Y	Y	7
Kranke, 2012[20]	Y	Y	Y	Y	Y	Y	Y	Y	Y	Y	Y	11
Martinez-Zapata, 2012[21]	U	Y	Y	N	Y	Y	Y	Y	Y	N	N	7
Peters, 2012[70]	U	N	Y	N	N	Y	Y	Y	Y	N	Y	6
Vermeulen, 2012[71] (CR: Vermeulen, 2005[113], Vermeulen, 2004[114])	Y	Y	Y	Y	Y	Y	Y	Y	Y	N	Y	10
Voigt, 2012[22]	U	Y	Y	Y	Y	Y	Y	Y	Y	Y	Y	10
Wilkinson, 2012 [23] (CR: Wilkinson, 1999[115], Wilkinson, 1998[116])	Y	Y	Y	N	Y	Y	Y	Y	Y	N	Y	9
Zarchi, 2012[72]	U	U	Y	N	N	Y	N	NA	N	N	N	2

Aziz, 2011[73] (CR: Aziz, 2010[117])	Y	Y	Y	Y	N	Y	Y	Y	Y	N	Y	9
Cruciani, 2011[24]	Y	Y	Y	Y	Y	Y	Y	Y	Y	Y	Y	11
Cullum, 2011[25]	Y	Y	Y	Y	Y	Y	Y	Y	Y	N	Y	10
Damiani, 2011[26]	U	Y	Y	N	N	Y	Y	Y	Y	N	Y	7
Dumville, 2011a[27]	Y	Y	Y	Y	Y	Y	Y	Y	Y	Y	Y	11
Dumville, 2012b[18]	Y	Y	Y	Y	Y	Y	Y	Y	Y	Y	Y	11
Hu, 2011[29]	U	U	Y	N	N	N	N	NA	N	N	Y	2
Hunt, 2011[79]	U	Y	Y	N	N	Y	Y	Y	Y	N	Y	7
Jull, 2011[30] (CR: Jull, 2007[118])	Y	Y	Y	Y	Y	Y	Y	Y	Y	Y	Y	11
Lima, 2011[74]	U	Y	Y	Y	Y	Y	Y	Y	Y	Y	Y	10
McGinnis, 2011[75]	Y	Y	Y	Y	Y	Y	Y	Y	Y	Y	Y	11
McInnes, 2011[76]	Y	Y	Y	Y	Y	Y	Y	Y	Y	Y	Y	11
Nelson, 2011a[77] (CR: Nelson, 2008[119])	U	N	Y	N	N	Y	Y	Y	N	N	Y	5
Nelson, 2011b[31]	Y	Y	Y	Y	Y	Y	Y	Y	Y	N	Y	10
Reddy, 2011[78]	U	U	Y	N	N	Y	Y	Y	Y	N	Y	6
Suissa, 2011[32]	U	U	Y	U	N	Y	N	NA	N	Y	Y	4

Voigt, 2011[33]	U	Y	Y	Y	N	N	Y	Y	Y	Y	Y	8
Carter, 2010[34]	U	U	Y	N	N	Y	Y	Y	Y	Y	Y	7
Chen, 2010[35]	U	Y	Y	N	N	Y	Y	Y	Y	N	Y	7
O'Meara, 2009a[44]	Y	Y	Y	Y	N	Y	Y	Y	Y	Y	Y	10
Pan, 2010[37]	U	U	Y	N	N	Y	Y	Y	N	N	Y	5
Vermeulen, 2010[80]	Y	Y	Y	Y	Y	Y	Y	Y	Y	N	Y	10
Villela, 2010[38]	U	U	Y	N	N	N	Y	Y	Y	N	Y	5
Xie, 2010[81]	U	U	Y	N	N	Y	Y	Y	Y	Y	Y	7
Amsler, 2009[39]	U	U	Y	N	N	Y	N	NA	N	N	Y	3
Jull, 2009[40]	Y	Y	Y	Y	Y	Y	Y	Y	Y	N	Y	10
Martinez-Zapata, 2009[42] (CR to Martinez-Zapata, 2012[21])	Y	Y	Y	Y	Y	Y	Y	Y	Y	N	Y	10
McGaughey, 2009[82]	U	N	Y	Y	N	Y	Y	Y	Y	N	N	6
Ministry of Health and Long-term Care, 2009a[43]	U	N	Y	Y	N	Y	Y	Y	Y	N	Y	7
Ministry of Health and Long-term Care, 2009b[83]	U	N	Y	Y	N	Y	Y	Y	Y	N	Y	7
Nelson,	Y	Y	Y	Y	Y	Y	Y	Y	Y	N	Y	10

2006[98] (CR: O'Meara, 2000[124])												
O'Meara, 2009b[45]	Y	Y	Y	Y	Y	Y	Y	Y	Y	Y	Y	11
O'Meara, 2010[36] (CR: O'Meara, 2009[121])	Y	Y	Y	Y	Y	Y	Y	Y	Y	Y	Y	11
Ramundo, 2009[85] (CR: Ramundo, 2008[123])	U	U	Y	N	N	Y	N	NA	N	N	N	2
Roukis, 2009[86]	U	U	Y	Y	N	Y	U	Y	N	N	Y	5
Barber, 2008[46]	U	Y	Y	Y	N	Y	Y	Y	N	N	N	6
Blozik, 2008[47]	U	U	Y	U	N	N	Y	N	Y	N	N	3
Flemming, 2008[48]	U	U	Y	Y	N	Y	Y	N	N	N	Y	5
Heyneman, 2008[87]	U	Y	Y	Y	N	N	Y	Y	Y	N	Y	7
Hinchliffe, 2008[88]	U	U	Y	N	N	Y	Y	Y	Y	N	Y	6
Howard, 2008[89]	Y	Y	Y	Y	N	Y	Y	Y	Y	N	Y	9
Langer, 2008[90]	Y	Y	Y	Y	Y	Y	Y	Y	Y	Y	Y	11
Lo, 2008[91]	U	Y	Y	Y	Y	Y	Y	Y	Y	Y	N	9
Moore, 2008[92]	U	Y	Y	Y	N	N	Y	Y	Y	N	N	6
Noble-Bell, 2008[93]	U	Y	Y	U	Y	Y	Y	Y	Y	N	N	7
Reddy,	U	U	Y	N	N	Y	Y	Y	N	N	Y	5

2008[94]												
Sadat, 2008[49]	U	U	Y	U	N	Y	N	NA	N	N	N	2
Ubbink, 2008a[95]	Y	Y	Y	Y	Y	Y	Y	Y	Y	N	Y	10
Ubbink, 2008b[96]	U	Y	Y	Y	N	Y	Y	Y	Y	N	N	7
van den Boogaard, 2008[97]	U	Y	Y	Y	N	Y	Y	Y	N	N	N	6
Chambers, 2007[50]	U	Y	Y	Y	N	Y	Y	Y	Y	N	N	7
Jones , 2007	U	Y	N	N	Y	Y	N	N	N	N	N	3
Lo, 2009[41]	U	Y	Y	Y	N	Y	Y	Y	Y	N	N	7
Palfreyman, 2007[52]	U	Y	Y	Y	Y	Y	Y	Y	Y	Y	Y	10
Nelson, 2009[84] (CR: Nelson, 2006[122])	U	Y	Y	Y	Y	Y	Y	Y	Y	Y	Y	10
O'Donnell, 2006[53]	Y	U	Y	N	N	N	Y	Y	Y	N	Y	6
Sari, 2006[54]	Y	N	Y	Y	N	Y	Y	Y	Y	N	Y	8
Bouza, 2005a[99]	U	U	Y	N	N	Y	Y	Y	Y	Y	Y	7
Bouza, 2005b[55]	U	Y	Y	Y	N	Y	Y	Y	Y	N	Y	8
Coleridge-Smith, 2005[56]	U	U	Y	Y	N	Y	Y	Y	N	Y	Y	7
Cruciani, 2005[57]	U	U	Y	Y	N	Y	Y	Y	Y	Y	N	7
Ho, 2005[58]	U	Y	Y	N	N	Y	Y	Y	Y	N	Y	7
Roeckl-Wiedmann,	Y	U	Y	Y	N	Y	Y	Y	Y	Y	N	8

2005[59]												
Schuren, 2005[100]	U	U	Y	Y	N	Y	Y	Y	Y	N	Y	7
Stratton, 2005[16]	U	U	Y	U	Y	Y	Y	Y	Y	Y	Y	8
Cullum, 2004[60]	Y	Y	Y	Y	Y	Y	Y	Y	Y	N	Y	10
Mwipatayi, 2004[101]	U	N	Y	N	N	N	Y	Y	N	N	N	3
Singh, 2004[61]	U	U	N	Y	N	Y	Y	Y	N	N	N	4
TenBrook, 2004[102]	U	U	N	N	N	Y	N	NA	N	Y	Y	3
Berliner, 2003[103]	U	N	Y	N	N	Y	N	NA	N	N	Y	3
Pham, 2003[104]	U	U	Y	Y	N	Y	Y	Y	N	N	Y	6
Wang, 2003[105]	U	U	N	N	N	Y	Y	Y	N	N	Y	4
Cullum, 2001a[62] (CR: Cullum, 2008[125])	U	Y	Y	Y	Y	Y	Y	Y	Y	Y	Y	10
Cullum, 2001b[62] (CR: Cullum, 2008[125])	U	Y	Y	Y	Y	Y	Y	Y	Y	Y	Y	10
Cullum, 2001c[62] (CR: Cullum, 2008[125])	U	Y	Y	Y	Y	Y	Y	Y	Y	Y	Y	10
Emergency Care Research Institute (ECRI), 2001[106]	Y	Y	Y	Y	Y	Y	Y	Y	Y	Y	Y	11

Evans, 2001[107]	Y	N	Y	Y	Y	Y	Y	Y	Y	N	Y	9
Lewis, 2001[108]	Y	Y	Y	Y	Y	Y	Y	Y	Y	N	Y	10
Moore, 2001[109]	U	Y	Y	N	N	N	Y	Y	N	N	N	4
O'Meara, 2001[110] (CR: O’Meara, 2000[124])	U	Y	Y	Y	N	Y	Y	Y	Y	N	N	7
Lucas, 2000[63]	U	Y	Y	Y	N	Y	Y	Y	Y	N	N	7
Bradley, 1999a[111]	U	N	Y	Y	N	Y	Y	Y	Y	Y	Y	8
Bradley, 1999b[64]	U	N	Y	Y	Y	Y	Y	Y	Y	Y	Y	9
Mason, 1999[112]	U	Y	Y	Y	N	Y	N	NA	N	N	N	4
Johannsen, 1998[65]	U	N	Y	N	N	Y	Y	Y	N	N	Y	5
Palfreyman, 1998[66]	U	Y	Y	N	N	Y	Y	Y	Y	N	N	6
Fletcher, 1997[67]	Y	Y	Y	Y	Y	Y	Y	Y	Y	N	Y	10
Note: NA = not applicable, N = no, U = unclear, Y = yes.											
