# Supplementary material for: Seeking effective interventions to treat complex wounds: an overview of systematic reviews
Source: BMC Med. 2015 Apr 22;13:89. doi: 10.1186/s12916-015-0288-5 (PMC4406332; doi:10.1186/s12916-015-0288-5)
Supplement: Additional file 6: — Non-meta-analysis results. Provides relevant findings from the included reviews without a meta-analysis. [file 12916_2015_288_MOESM6_ESM.pdf]

Non-meta-analysis results				
**Intervention**	**Review (# of studies included)**	**Comparator**	**Conclusion**	**Conclusion**
Venous leg ulcers – time to healing or rate of healing (1 review)				
Applied freeze-dried keratinocyte lysate (topical)	Nelson, 2011a[77] (CR: Nelson, 2008[119]) (1)	Vehicle or usual care	Topically applied freeze-dried keratinocyte lysate seems no more effective at increasing healing rates at 24 weeks	Neutral
Collagenase (topical)	Nelson, 2011a[77] (CR: Nelson, 2008[119]) (31)	Usual care or versus each other (debriding agents)	Results are inconclusive due to incomplete reporting of the studies.	Indeterminate
Compression stockings	Nelson, 2011a[77] (CR: Nelson, 2008[119]) (19)	Compression bandages	Compression stockings may be more effective at increasing healing rates and reducing mean time to healing in people with venous leg ulcers.	Positive
Flavonoids + compression	Nelson, 2011a[77] (CR: Nelson, 2008[119]) (14)	Compression	We don't know whether flavonoids plus compression are more effective than compression alone at increasing ulcer healing rates	Indeterminate
Intermittent pneumatic compression + compression	Nelson, 2011a[77] (CR: Nelson, 2008[119]) (4)	Compression stockings or bandages alone	We don't know whether adding pneumatic compression to compression stockings is more effective than stockings or bandages alone at increasing healing rates	Indeterminate
Larval therapy	Nelson, 2011a[77] (CR: Nelson, 2008[119]) (1)	Hydrogel	Compared with hydrogel, we don't know whether larval therapy is more effective at improving time to ulcer healing in people with venous leg ulcers	Indeterminate
Laser therapy	Nelson, 2011a[77] (CR: Nelson,	Sham treatment	Compared with sham or control treatment, we don't know whether low-level	Indeterminate

	2008[119]) (7)		laser treatment is more effective at increasing ulcer healing rates at 4 weeks to 9 months	
Leg ulcer clinics, wound care program	Nelson, 2011a[77] (CR: Nelson, 2008[119]) (5)	Usual care	Compared with usual care we don't know whether leg ulcer clinics are more effective at increasing ulcer healing rates	Indeterminate
Multi-layer elastomeric high –compression	Nelson, 2011a[77] (CR: Nelson, 2008[119]) (12)	Short-stretch bandages or Unna's boot/paste-based systems	Multi-layer elastomeric high-compression bandages seem no more effective than short-stretch bandages at increasing healing rates, but may reduce time to healing	Neutral
Platelet-derived growth factor (oral)	Nelson, 2011a[77] (CR: Nelson, 2008[119]) (2)	Placebo	Compared with placebo, we don't know whether platelet-derived growth factors are more effective at increasing ulcer healing rates	Indeterminate
Rutosides (oral)	Nelson, 2011a[77] (CR: Nelson, 2008[119]) (3)	Placebo	Compared with placebo, we don't know whether oral rutosides alone or with compression are more effective than placebo at increasing ulcer healing rates at 6 to 12 weeks	Indeterminate
Semi-occlusive dressings: foam, film, hyaluronic acid-derived dressings, collagen, cellulose, or alginate	Nelson, 2011a[77] (CR: Nelson, 2008[119]) (16)	Simple low-adherent dressings, in the presence of compression	Compared with simple low-adherent dressings, semi-occlusive dressings (foam, film, hyaluronic acid-derived dressings, collagen, cellulose, or alginate) may be no more effective than simple low-adherent dressings (such as paraffin-tulle or knitted viscose dressings) at increasing wound healing rates in the presence of compression	Neutral
Single-layer non-elastic system stockings	Nelson, 2011a[77]	Multi-layer non-elastic system	We don't know how single-layer and multi-	Indeterminate

	(CR: Nelson, 2008[119]) (1)		layer non-elastic systems compare at increasing healing rate.	
Stockings: multi-layer elastic system, multi-layer elastomeric (or non-elastomeric) high-compression regimens	Nelson, 2011a[77] (CR: Nelson, 2008[119]) (10)	Multi-layer elastic system, multi-layer elastomeric (or non-elastomeric) high-compression regimens, four-layer compression bandages, other multi-layer high-compression bandages	Multi-layer elastomeric high-compression regimens compared with each other, four-layer compression bandages and other multi-layer high-compression bandages may be equally effective at increasing healing rates	Neutral
Sulodexide (oral) + compression	Nelson, 2011a[77] (CR: Nelson, 2008[119]) (4)	Compression	Compared with compression alone, oral sulodexide plus compression is more effective at increasing healing rates at 2 to 3 months	Positive
Thromboxane α2 antagonists (oral)	Nelson, 2011a[77] (CR: Nelson, 2008[119]) (1)	Placebo	Compared with placebo, we don't know whether oral thromboxane α2 antagonists are more effective at increasing ulcer healing rates	Indeterminate
Topical negative pressure (vacuum-assisted closure)	Nelson, 2011a[77] (CR: Nelson, 2008[119]) (1)	Usual care	Compared with usual care, topical negative pressure (vacuum-assisted closure) may be more effective than conventional wound care techniques at reducing time to complete healing in people with venous or arteriovenous ulcers of at least 6 months' duration	Positive
Ultrasound	Nelson, 2011a[77] (CR: Nelson, 2008[119]) (2)	Standard care	Compared with standard care, ultrasound is no more effective than standard care at reducing time to healing at 12 weeks and increasing the proportion of people with healed ulcers at 12 months	Neutral
<u>Venous leg ulcers – ulcer healing (5 reviews)</u>				
Compression bandages,	Nelson,	No compression	Compression bandages	Positive

stockings	2011a[77] (CR: Nelson, 2008[119]) (7)		and stockings heal more ulcers compared with no compression.	
	Nelson, 2011a[77] (CR: Nelson, 2008[119]) (1)	Intermittent pneumatic compression	The RCT is too small to draw a reliable conclusion.	Indeterminate
Electromagnetic therapy	Aziz, 2011[73] (CR: Aziz, 2010[117]) (3)	Sham electromagnetic therapy	2/3 trials positive for electromagnetic therapy vs sham electromagnetic therapy.	Positive*
Hydrocolloid (occlusive) dressings + compression	Nelson, 2011a[77] (CR: Nelson, 2008[119]) (23)	Simple dressings	Compared with simple dressings, hydrocolloid dressings are no more effective than simple low-adherent dressings at increasing ulcer healing rates in people receiving compression	Neutral
Intermittent pneumatic compression (Flowtron, sequential gradient Jobst extremity pump)	Berliner, 2003[103] (8)	Other compression devices, standard wound care (Setopress, Unna boot, debridement, ulcer cleansing, compression stocking, continued compression bandage)	3/8 studies showed positive for intermittent pneumatic compression but available data cannot be relied on to inform the optimal choice of compression therapy or optimal protocol.	Indeterminate
Maggot debridement therapy	Zarchi, 2012[72] (7)	Hydrogel, hydrocolloid, conventional therapy (saline gauze)	Larval therapy as being significantly more effective than hydrogel or a mixture of conventional therapy modalities, including hydrocolloid, hydrogel and saline moistened gauze.	Positive
Mesoglycan (topical)	Nelson, 2011a[77] (CR: Nelson, 2008[119]) (1)	Plant-based extract, topical	Compared with plant-based extract, we don't know how topical mesoglycan (a profibrinolytic agent) and plant-based extract compare at increasing ulcer healing.	Indeterminate
Skin grafts	Nelson, 2011a[77] (CR:	Usual care or vs each other	Different types of skin grafts compared with other treatments for	Indeterminate

	Nelson, 2008[119]) (1)		leg ulcers, we don't know how different types of skin grafts and other treatments for leg ulcers compare at increasing healing of venous ulcers	
Superficial venous surgery	Howard, 2008[89] (54)	Conservative compression therapy	Surgery results in a less impressive reduction in ulcer recurrence in patients. These patients appear to still benefit from surgery due to the hemodynamic and clinical benefits that result.	Positive*
Venous leg ulcers – proportion of patients with healed wounds (2 reviews)				
Antimicrobial (topical)	Nelson, 2011a[77] (CR: Nelson, 2008[119]) (1)	Placebo or usual care	Compared to placebo or usual care, topical antimicrobial agents may be no more effective at increasing the proportion of people with completely healed ulcers.	Neutral
Autologous platelet lysate (topical)	Nelson, 2011a[77] (CR: Nelson, 2008[119]) (1)	Placebo	Compared with placebo, topically applied autologous platelet lysate seems no more effective at increasing the proportion of people with healed ulcers at 9 months	Neutral
Calcitonin gene-related peptide (topical)	Nelson, 2011a[77] (CR: Nelson, 2008[119]) (1)	Placebo	Compared with placebo, calcitonin gene-related peptide plus vasoactive intestinal polypeptide seems no more effective at increasing the proportion of people with healed ulcers at 12 weeks	Neutral
Cultured allogenic bilayer skin replacement	Nelson, 2011a[77] (CR: Nelson, 2008[119]) (2)	Non-adherent dressing	Cultured allogenic bilayer skin replacement (containing both epidermal and dermal components) seems more effective at increasing the proportion of healed ulcers.	Positive

Granulocyte-macrophage colony-stimulating factor (peri-ulcer injection)	Nelson, 2011a[77] (CR: Nelson, 2008[119]) (1)	Placebo	Recombinant human granulocyte-macrophage colony-stimulating factors (rHuGM-CSF) are more effective at increasing the proportion of people with completely healed ulcers at 13 weeks	Positive
Multi-layer elastomeric high-compression bandages	Nelson, 2011a[77] (CR: Nelson, 2008[119]) (4)	Single-layer bandages	Multi-layer elastomeric high-compression bandages compared with single-layer bandage multi-layer compression bandages are more effective at increasing the proportion of people with healed ulcers	Positive
Pentoxifylline (oral) + compression)	Nelson, 2011a[77] (CR: Nelson, 2008[119]) (7)	Placebo	Compared with placebo, oral pentoxifylline plus compression is more effective at increasing the proportion of people with healed ulcers at 8 to 24 weeks.	Positive
Prostaglandin E1 (IV)	Nelson, 2011a[77] (CR: Nelson, 2008[119]) (1)	Placebo	Compared with placebo, intravenous prostaglandin E1 may be more effective at improving the number of healed ulcers at 120 days	Positive
Single-layer non-elastic system stockings	Nelson, 2011a[77] (CR: Nelson, 2008[119]) (2)	Multi-layer elastic system	Unclear whether single-layer is more effective at increasing the proportion of limbs with complete healing of ulcers	Indeterminate
Subfascial endoscopic perforator surgery	Tenbrook, 2004[102] (20)	Unclear	Overall, after surgical treatment including subfascial endoscopic perforator surgery, with or without concomitant superficial venous ablation, ulcers in 88% of limbs healed. Ulcers recurred in 13%, at mean time of 21 months.	Positive

Superficial vein surgery	Nelson, 2011a[77] (CR: Nelson, 2008[119]) (2)	No surgery or versus surgery plus skin grafting in the presence of compression	Perforator ligation compared with no surgery or surgery plus skin grafting, we don't know whether perforator ligation is more effective at increasing the proportion of ulcers healed at 1 year or at reducing time to ulcer healing	Indeterminate
Systemic mesoglycan (IM, oral) + compression	Nelson, 2011a[77] (CR: Nelson, 2008[119]) (1)	Placebo + compression	Systemic mesoglycan plus compression seems more effective at increasing the proportion of people with healed ulcers.	Positive
<u>Mixed arterial/venous leg ulcers – ulcer healing (6 reviews)</u>				
Ciprofloxacin (oral), trimethoprim (oral), polynoxilin (topical), dimethyl sulfoxide (topical), mupirocin (topical), povidone-iodine (topical) with hydrocolloid, silver cream or impregnated dressing, levamisole (oral), benzoyl peroxide (topical), AZAC cream, eosin and chloroxylenol spray, hexachlorophane lotion, oxyquinoline lotion, gentian violet 0.1% with dibutyryl adenosine (topical), amoxylcillin (oral), clindamycin (oral), chlorhexadine (topical), cefotoxin, metronidazole, gentamicin, Eusol pack	O'Meara, 2001[110] (CR: O'Meara, 2000[124]) (22)	Placebo, allopurinol (topical), hydrocolloid dressing, another antibiotic, collagen gel, cetrimide lotion, clavulanic acid, hydrogel dressing, silastic foam dressing	Results do not support routine use of systemic antibiotics for leg ulcers or diabetic foot ulcers without acute infection, but they may be useful as an adjunct to surgery for pilonidal sinuses.	Neutral
Electromagnetic therapy	McGaughey, 2009[82] (11)	A sham or a different intensity of pulsed electromagnetic therapy. No pulsed electromagnetic therapy or other treatments.	For venous ulcers, there is strong evidence that pulsed electromagnetic therapy is more effective than sham pulsed electromagnetic therapy. For pressure and plantar ulcers, there is moderate evidence that pulsed electromagnetic therapy, in combination with	Positive

			conventional wound care is better than conventional wound care alone.	
Honey (topical)	Mwipatayi, 2004[101] (2)	Phenytoin, topical, commercial wound dressings or systemic and topical antibiotics	Inconclusive due to trials differences.	Indeterminate
Ketanserin ointment, 2% (topical)	Nelson, 2009[84] (CR: Nelson, 2006[122]) (1)	Vehicle (polyethylene glycol)	The trial was too small and for too short a follow-up period to be able to determine any difference in healing rates	Indeterminate*
Primary nursing delivery, team members received training in leg ulcer management; standardized treatment from a wound care team comprising trained community pharmacists and nurses - a standardized protocol along with training was provided to team members	Ministry of Health and Long-term Care, 2009b[83] (2)	Usual care (no wound treatment protocol was used)	The evidence supports that managing chronic wounds with a multidisciplinary wound care team significantly increases wound healing	Positive
Silver releasing dressing	Vermeulen, 2010[80] (3)	Dressing with silver, different dosages, no silver, other antiseptics	The data from these trials show that silver-containing foam dressings did not significantly increase complete ulcer healing as compared with standard foam dressings or best local practice after up to four weeks of follow-up.	Neutral*
Diabetes foot/leg ulcers – wound area/size reduction (2 reviews)				
Hyperbaric oxygen therapy (systemic + usual care)	Hunt, 2011[79] (CR: Hunt, 2009[120]) (1)	Usual care	Hyperbaric oxygen plus usual care may be no more effective at promoting ulcer healing.	Neutral
Stem cell therapy	Hinchliffe, 2008[88] (2)	Saline, conventional treatment	2/2 studies positive for stem cell therapy vs placebo.	Positive
Diabetes foot/leg ulcers – time to healing or rate of healing (3 reviews)				
Human skin equivalent	Hunt, 2011[79] (CR: Hunt, 2009[120]) (1)	Saline-moistened gauze	Study positive for human skin equivalent vs saline-moistened gauze	Positive
Human cultured dermis	Hunt,	Usual care	Human cultured	Neutral

	2011[79] (CR: Hunt, 2009[120]) (1)		dermis substitute plus usual care is no more effective at increasing ulcer healing rates vs usual care	
Laser therapy and complex intervention	Hinchliffe, 2008[88] (1)	NR	Couldn't draw conclusions from study.	Indeterminate
Platelet derived growth factors (topical)	Hunt, 2011[79] (CR: Hunt, 2009[120]) (6)	Placebo	4/6 studies positive for topical growth factors vs placebo	Positive
Pressure off-loading, felted foam	Hunt, 2011[79] (CR: Hunt, 2009[120]) (1)	Pressure off-loading (pressure-relief half shoe)	No significant difference between felted foam vs pressure-relief half shoe	Neutral
Pressure off-loading, total contact or non-removable cast	Hunt, 2011[79] (CR: Hunt, 2009[120]) (1)	Traditional dressing changes	Study positive for pressure off-loading vs traditional dressing changes	Positive
Stem cell therapy	Hinchliffe, 2008[88] (1)	Saline	1/1 study neutral (no significant difference) for stem cell vs saline.	Neutral
Skin grafts	Hinchliffe, 2008[88] (1)	Split thickness graft	1/1 study neutral (not significant) for skin grafts vs controls.	Neutral
Topical negative pressure	Ubbink, 2008a[95] (7)	Hydrocolloid wound gel and gauze dressings, chlorophyllin copper complex /Papain-urea topical, saline gauze, hydrogels, alginates, compression bandages, ringers soaked bandages	Topical negative pressure does not significantly increase the healing rate of chronic wounds compared with comparators.	Neutral*
<u>Diabetes foot/leg ulcers – ulcer healing (10 reviews)</u>				
Alginate, foam, hydrogel, hydrocolloid dressings	Mason, 1999[112] (10)	Sterile gauze dressings, other dressings	Inconclusive	Indeterminate
Alginate, hydrogel, hydrocellulase, semi-permeable membrane dressings	Hinchliffe, 2008[88] (7)	Saline-moistened gauze, wet/dry sterile gauze	2/2 studies showed no difference between alginate vs saline-moistened gauze. 1/3 studies were positive for hydrogel vs saline-moistened gauze. 1/1 study positive for hydrocellulase vs saline-moistened gauze. 1/1 study positive for semi-permeable membrane vs saline-moistened	Neutral

			gauze.	
Amoxicillin + clavulanic acid (oral), ofloxacin, imipenem/cilastatin, ampicillin/sulbactam (IV)	Mason, 1999[112] (4)	Placebo, other antibiotics	Inconclusive	Indeterminate
Antibiotics, choice based on bone biospsy (IV, oral)	Peters, 2012[70] (1)	NR	Inconclusive	Indeterminate
Ayurvedic preparations (oral + topical)	Nelson, 2006[98] (CR: O'Meara, 2000[124]) (1)	Standard care	The majority of trials were underpowered and too dissimilar to be pooled. There was no strong evidence for recommending any particular antimicrobial agent for the prevention of amputation, resolution of infection or ulcer healing.	Indeterminate*
Clindamycin, fluoroquinolone, rifampicin, amoxicillin/clavulanic acid (oral, topical) +/- surgical intervention	Lima, 2011[74] (8)	No controls; all observational studies	There is insufficient evidence to support early use of broad-spectrum antibiotics against methicillin-resistant Staphylococcus aureus to promote healing of diabetic ulcers, since antibiotic resistance may develop from such treatment.	Indeterminate*
Compression	Hinchliffe, 2008[88] (1)	Placebo	1/1 study positive for compression vs placebo	Positive
Cultured human dermis	Mason, 1999[112] (2)	Standard wound care, conventional care	Inconclusive	Indeterminate
Dressings + debridement (hydrogel)	Hunt, 2011[79] (CR: Hunt, 2009[120]) (4)	Other debridement techniques (hydrogel), standard wound care	3/4 studies positive for hydrogel vs standard wound care. Indeterminate study between hydrogel purilon vs hydrogel intrasite.	Positive
Early surgical intervention + antibiotics	Peters, 2012[70] (2)	Systemic antibiotics	Hard to draw any conclusions from these data	Indeterminate
Electrical stimulation	Hinchliffe, 2008[88] (2)	NR	1/2 studies positive for electrical stimulation	Neutral
Endovascular or open bypass revascularization surgery of an ulcerated foot	Hinchliffe, 2012[69] (49)	NR	There were insufficient data to recommend one method of revascularization over	Indeterminate

			another. Of the 49 studies cited in this review, 46 are case series and have no control group. Of the remaining three studies with a control group: no revascularization was noted.	
Foot care clinic interventions	Hunt, 2011[79] (CR: Hunt, 2009[120]) (1)	Usual care	Indeterminate	Indeterminate
Granulocyte-colony stimulating factor (SC)	Mason, 1999[112] (1)	Placebo	Initial result indicates that granulocyte-colony stimulating factor treatment should receive more extensive evaluation	Indeterminate
	Nelson, 2006[98] (CR: O'Meara, 2000[124]) (4)	Standard care, placebo	The majority of trials were underpowered and too dissimilar to be pooled. There was no strong evidence for recommending any particular antimicrobial agent for the prevention of amputation, resolution of infection or ulcer healing.	Indeterminate*
	Peters, 2012[70] (5)	Usual care	Results of these five studies are inconsistent and provide no clear evidence on which intervention is more effective	Indeterminate
Growth factors (topical)	Hinchliffe, 2008[88] (8)	Placebo, saline-moistened gauze	4/8 studies positive for growth factors vs placebo	Neutral
Hydrogel, cadexomer iodine ointment, dressings, larval therapy, sugar (topical) systemic antibiotics	Nelson, 2006[98] (CR: O'Meara, 2000[124]) (5)	Saline gauze, dry gauze, antibiotics, standard care, streptokinase, 2% eosin and 0.3% chloroxylenol spray, gauze and chlorhexidine, sugar dressings	The majority of trials were underpowered and too dissimilar to be pooled. There was no strong evidence for recommending any particular antimicrobial agent for the prevention of amputation, resolution of infection or ulcer healing.	Indeterminate*
Hyperbaric oxygen	Hinchliffe,	Standard care	5/6 studies positive for	Positive

therapy	2008[88] (6)		hyperbaric oxygen therapy vs standard care.	
	Hunt, 2011[79] (CR: Hunt, 2009[120]) (2)	Usual care	2/2 studies positive for hyperbaric oxygen therapy vs usual care.	Positive
	Mason, 1999[112] (2)	Clinical management, standard care	Studies show no significant differences between hyperbaric oxygen therapy and standard care, clinical management	Neutral
	Peters, 2012[70] (1)	NR	Inconclusive	Indeterminate
Hyperbaric oxygen therapy (systemic + usual care)	Hunt, 2011[79] (CR: Hunt, 2009[120]) (1)	Usual care	Hyperbaric oxygen plus usual care may be no more effective at promoting ulcer healing.	Neutral
Imipenem/cilastatin, cefazolin, Ampicillin/sulbactam, Linezolid, Piperacillin/tazobactam. Amoxycillin + clavulanic acid, clindamycin hydrochloride (oral), pexiganan cream	Nelson, 2006[98] (CR: O'Meara, 2000[124]) (13)	Piperacillin/clindamycin, ceftriaxone, cefoxitin, amoxicillin clavulanate, placebo, cephalexin, ofloxacin, standard care.	There was no strong evidence for recommending any particular antimicrobial agent for the prevention of amputation, resolution of infection or ulcer healing. Topical pexiganan cream may be as effective as oral antibiotic treatment with ofloxacin for the resolution of local infection.	Neutral*
Ketanserin (oral, topical)	Mason, 1999[112] (2)	Placebo, saline	Inconclusive	Indeterminate
Larval therapy	Hinchliffe, 2008[88] (2)	NR	2/2 studies positive for larval therapy	Positive
Lyophilized collagen, platelets and derived products (topical)	Hinchliffe, 2008[88] (4)	Hyaluronic acid-medicated gauze, saline, placebo	1/1 study positive for lyophilized collage vs hyaluronic acid-medicated gauze. 3/4 studies positive for platelets and derived products.	Positive
Magnet and normothermic therapy	Hinchliffe, 2008[88] (3)	Saline	2/2 no significant differences (neutral) for normothermic therapy. No significant differences between magnet therapy and control.	Neutral

Patient education	Hunt, 2011[79] (CR: Hunt, 2009[120]) (5)	Usual care	Indeterminate	Indeterminate
	Mason, 1999[112] (1)	Usual care	The education intervention contains a “scare-tactic” component and it is unclear whether this approach is generalizable. Method merits evaluation in other contexts.	Indeterminate
Percutaneous flexor tenotomy	Roukis, 2009[86] (2)	All are case series so there aren't any control groups	Inconclusive due to poor methodologies of studies.	Indeterminate
Procaine + polyvinylpyrrolidone (IM)	Peters, 2012[70] (1)	NR	No significant difference between groups	Neutral
Resection of the chronic wound	Hinchliffe, 2008[88] (4)	Conservative or usual treatment	2/4 studies positive for surgery vs conservative or usual treatment.	Neutral
Sharp debridement	Hinchliffe, 2008[88] (1)	NR	The evidence of benefit of sharp debridement is not strong and is based on a single study comprising a subgroup analysis of cases from an RCT of another intervention. Healing at 12 weeks was more likely following a more vigorous debridement.	Indeterminate
Skin grafts	Hinchliffe, 2008[88] (5)	Saline moistened gauze, conventional treatment, standard care	4/5 studies positive for skin grafts vs standard care, saline moistened gauze, conventional treatment	Positive
Stem cell therapy	Hinchliffe, 2008[88] (2)	Standard/conventional treatment	2/2 positive for stem cell therapy vs standard/conventional treatment	Positive
Superoxidized water and soap, povidone iodine (topical)	Peters, 2012[70] (2)	Other topical disinfectants	Inconclusive	Indeterminate
Therapeutic footwear	Hunt, 2011[79] (CR: Hunt, 2009[120]) (2)	Usual footwear	Indeterminate	Indeterminate
Thrombin-induced	Mason,	Placebo	These findings need	Indeterminate

human platelet growth factor, recombinant platelet derived growth factor, recombinant basic fibroblast growth factor, arginine-glycine-aspartic acid peptide matrix (topical)	1999[112] (6)		confirmation from further trials	
Topical negative pressure	Hinchliffe, 2008[88] (3)	Saline moistened gauze, standard care	3/3 studies positive for topical negative pressure vs saline moistened gauze, standard care.	Positive
Negative pressure therapy	Noble-Bell, 2008[93] (4)	Dressings: alginates, hydrocolloids, foams or hydrogels, saline gauze, moist dressing	Negative pressure therapy is more effective than conventional dressings, but quality of the studies were weak.	Positive
	Xie, 2010[81] (10)	Standard wound dressing, wet-to-dry dressings, advanced moist wound therapy and stand moist gauze dressing	Consistent evidence of the benefit of negative pressure therapy compared with control treatments.	Positive
Total contact casting	Mason, 1999[112] (1)	Standard treatment	Preliminary finding from this one small trail suggests further investigation of this intervention is appropriate	Indeterminate
Ultrasound	Hinchliffe, 2008[88] (1)	Sham therapy	No significant differences for ultrasound vs sham	Neutral
Zinc oxide tape	Hinchliffe, 2008[88] (1)	Hydrocolloid dressing	1 study positive for zinc oxide vs hydrocolloid.	Positive
<u>***Pressure ulcers – wound area/size reduction (3 reviews)***</u>				
Air-fluidised support	Reddy, 2011[78] (4)	Standard care	Compared with standard care, air-fluidised supports may be more effective than standard care (alternating-pressure mattresses, regular changes of position, sheepskin, gel pads, or limb protectors) at healing established pressure ulcers.	Positive
Alternating pressure mattress, low-air-loss mattress, air-fluidised mattress	Reddy, 2008[94] (8)	Fluid mattress overlay, air and foam mattress, specialized foam mattress overlay, foam overlay, alternating pressure	No clear evidence favored one support surface over another.	Neutral

		mattress, alternating pressure mattress covered with foam		
Collagenase	Reddy, 2011[78] (3)	Other debridement agents	Unclear whether any one debriding agent is consistently more effective than the other debriding agents at healing pressure ulcers.	Indeterminate
Collagenase, hydrogel dressings	Reddy, 2008[94] (6)	Pain-urea-chlorophyllin copper, fibrinolysin or deoxyribonuclease, hydrocolloid, moist saline gauze, dextranomer, sugar and egg white	No other dressing was superior to alternatives. Little evidence supports the use of a specific support surface or dressing over other alternatives	Neutral
Electric current, electromagnetic therapy	Reddy, 2008[94] (5)	Placebo	There is little evidence to support adjunctive therapies compared with standard care.	Neutral
Foam, calcium alginate, radiant heat dressing, dextranomer powder dressings	Reddy, 2008[94] (4)	Foam with wound-contact layer, dextranomer, topical, alginate dressing, moist saline gauze	No other dressing was superior to alternatives. Little evidence supports the use of a specific support surface or dressing over other alternatives.	Neutral
Hydrocolloid dressings	Heyneman, 2008[87] (29)	Saline gauze, foam dressing, hydrogels, less-contact layers, topical enzymes, povidine gauze, pheytoin, biosynthetics, radiant heat therapy (wound wrapped in polyurethane film), sequential use of hydrocolloids and alginates dressing	Hydrocolloids are more effective than gauze dressings for the reduction of the wound dimensions.	Positive
Hydrocolloid, hydrogel wafer, hydrogel, occlusive polyurethane, transparent moisture-permeable dressings	Reddy, 2008[94] (10)	Hydrocolloid dressing, calcium alginate dressing, polyurethane foam dressing, dextranomer, moist saline gauze	No other dressing was superior to alternatives. Little evidence supports the use of a specific support surface or dressing over other alternatives.	Neutral
Hydrogel, cadexomer iodine, semelil gel, radiant heat, zinc salt spray, aluminum hydroxide, vitamin A ointment, streptokinase-streptodornase, dialysate,	Reddy, 2008[94] (10)	Povidoine-iodine gauze, placebo spray and ointment, zinc oxide, standard care, moist saline gauze, saline	No other dressing was superior to alternatives. Little evidence supports the use of a specific support surface or dressing over other	Neutral

topical insulin, moist saline gauze and whirlpool, semelil dressings			alternatives.	
Low level laser therapy, laser and standard care	Reddy, 2008[94] (2)	Standard care, ultrasound and UV-C and standard care	There is little evidence to support adjunctive therapies compared with standard care.	Neutral
Polarized light, monochromatic light and cadexomer iodine or hydrocolloid	Reddy, 2008[94] (2)	Standard care, cadexomer iodine or hydrocolloid	There is little evidence to support adjunctive therapies compared with standard care.	Neutral
Ultrasound	Reddy, 2008[94] (1)	Placebo ultrasound	There is little evidence to support adjunctive therapies compared with standard care.	Neutral
Vacuum therapy	Reddy, 2008[94] (2)	Moist gauze, cadexomer iodine, topical, papain-urea-chlorophyllin copper, topical	There is little evidence to support adjunctive therapies compared with standard care.	Neutral
Vitamin C and ultrasound, consistent wound care and controlled nutritional support, vitamin C, zinc sulfate	Reddy, 2008[94] (4)	Vitamin C and placebo ultrasound, standard care and standard diet, placebo	Nutritional supplement studies showed mixed results.	Neutral
Pressure ulcers - time to healing or rate of healing (3 reviews)				
Ascorbic acid, high-protein diet, concentrated, fortified, collagen protein hydrolysate supplement, disease-specific nutrition treatment	Reddy, 2011[78] (8)	Control (low dose or no supplements)	Unclear whether nutritional supplements are more effective than control at increasing healing of pressure ulcers	Indeterminate
Amorphous hydrogel dressing derived from Aloe vera wound dressings	Dat, 2012[68] (1)	Saline gauze dressing	No significant difference between interventions. The poor quality of the included trials indicates that the trial results must be viewed with extreme caution as they have a high risk of bias.	Indeterminate*
Electromagnetic therapy, low-intensity direct current, negative-polarity and positive-polarity electrotherapy, and alternating-polarity electrotherapy	Reddy, 2011[78] (7)	Sham, standard treatment	Unclear whether electrotherapy is more effective than sham electrotherapy or standard care at healing pressure ulcers as we found insufficient evidence.	Indeterminate
Hydrocolloid dressings	Reddy, 2011[78] (1)	Gauze soaked in saline, hypochlorite, or povidone iodine	Unclear whether hydrocolloid dressings are more effective at healing pressure ulcers	Indeterminate

			vs gauze soaked in saline, hypochlorite or povidone iodine.	
Low-air-loss beds	Reddy, 2011[78] (5)	Standard beds, standard care	Compared with standard beds or standard care, we don't know whether low-air-loss beds are more effective at increasing pressure ulcer healing.	Indeterminate
Low level laser therapy	Reddy, 2011[78] (2)	Sham treatment, standard care	Unclear whether laser treatment is more effective than standard care at increasing pressure ulcer healing.	Indeterminate
Low-tech constant-low-pressure supports	Reddy, 2011[78] (1)	Other low-tech constant-low-pressure supports	Unclear whether a layered-foam replacement mattress is more effective than a water mattress at increasing healing of pressure ulcers.	Indeterminate
Phenytoin ointment (topical)	Reddy, 2011[78] (4)	Hydrocolloid/standard dressings or antibiotic ointment	Unclear whether topical phenytoin ointment is more effective at increasing pressure ulcer healing vs hydrocolloid/standard dressings or antibiotic ointment.	Indeterminate
Seat cushions	Reddy, 2011[78] (3)	Seat cushions, standard care	Unclear whether seat cushions are more effective than standard care at reducing time to complete healing, or whether different seat cushions differ in effectiveness at increasing pressure ulcer healing.	Indeterminate
Topical negative pressure	Reddy, 2011[78] (2)	Control (gauze soaked in Ringer's solution or a regimen of three gel products)	Unclear whether topical negative pressure is more effective than gauze soaked in Ringer's solution or a regimen of three gel products at increasing healing of pressure ulcers.	Indeterminate
Triple antibiotic ointment, active cream dressings	Reddy, 2008[94] (2)	Placebo, hydrocolloid, phenytoin suspension	Little evidence supports the use of a specific support surface or dressing over other alternatives.	Neutral

Ultrasound	Reddy, 2008[94] (1)	Placebo ultrasound	There is little evidence to support adjunctive therapies compared with standard care.	Neutral
Pressure ulcers – ulcer healing (11 reviews)				
Air-fluidised beds	Emergency Care Research Institute, 2001[106] (10)	Conventional therapies (including: alternating pressure pads, air support mattresses, water mattresses and high-density foam pads	Not conclusive, with none demonstrating statistically significant differences between Group 2 support surfaces and foam mattresses in complete ulcer healing.	Indeterminate*
Air-fluidised beds, air suspension beds, foam replacement mattress	McInnes, 2011[76] (18)	Conventional treatment, 2-hourly turns, heel and elbow protectors, alternating-pressure mattresses, low-air-loss mattress, wheelchair seat cushion, foam mattress overlay, flat-based bed with a pressure relieving/redistributing mattress/cushion, convoluted foam mattress overlay, standard care, fluid overlay mattress	There is no conclusive evidence about the superiority of any support surface for the treatment of existing pressure ulcers.	Neutral*
Alternating pressure surfaces	Reddy, 2011[78] (5)	Standard care, other care	Unclear whether alternating-pressure surfaces are more effective than standard care at healing pressure ulcers, or whether any one alternating-pressure surface is consistently more effective than all the others.	Indeterminate
Alternating pressure surfaces (alternating pressure mattress + pressure relief cushion)	McGinnis, 2011[75] (1)	Pegasus Cairwave alternating mattress replacement (1 in 3 alternating cycle) and Proactive cushion	Indeterminate	Indeterminate*
Ascorbic acid, zinc sulphate	Langer, 2008[90] (4)	10 mg ascorbic acid , high protein, placebo	It was not possible to draw any firm conclusions on the effect of enteral and parenteral nutrition on the prevention and treatment of pressure ulcers.	Indeterminate*
Collagen protein, standard hospital diet and high protein, standard hospital diet and high protein and zinc and	Reddy, 2008[94] (3)	Placebo, standard hospital diet, standard hospital diet + high protein + zinc + arginine + vitamin C + antioxidants	Nutritional supplement studies showed mixed results.	Neutral

arginine and vitamin C				
Collagenase (topical)	Ramundo, 2009[85] (CR: Ramundo, 2008[123]) (12)	Placebo, hydrogel, silver sulfadiazine, autolysis, papin-urea ointment, trypsin, fibrinolysin/DNAse ointment	Collagenase ointment is a safe and effective choice for debridement of cutaneous ulcers.	Positive
Fibroblast-derived dermal replacement	Reddy, 2008[94] (1)	Standard care	Study positive for skin substitute vs standard care. However, the incremental benefit of this biological agent over less expensive standard wound care remains uncertain.	Positive
Hydrocolloid, polyurtheate, dextranomer, hydrogel, polyhydroxyethyl metahcrylate, amino acid copolymer dressings	Bouza, 2005a[99] (21)	Other advanced dressings, conventional dressings (Saline gauze, Gauze Dakin's solution, Poridone-iodine guaze, Paraffin guaze, Collagen, Hydrocolloid Alginate)	Comparisons showed greater efficacy of hydrocolloid dressings but failed to confirm advantages of other advanced dressings compared with conventional ones	Neutral
Low-air-loss mattress, alternating pressure mattress, air-fluidised mattress	Reddy, 2008[94] (1)	Specialized foam mattress overlay, alternating pressure mattress	No clear evidence favored one support surface over another.	Neutral
Phenytoin solution, antibiotics dressings	Reddy, 2008[94] (2)	Antibiotic ointment, honey dressing	Little evidence supports the use of a specific support surface or dressing over other alternatives	Neutral
Protease-modulating matrix, recombinant platelet-derived growth factor BB, nerve growth factor, transforming growth factor beta, granulocyte-macrophage/colony stimulating factor, basic fibroblast growth factor (topical)	Reddy, 2008[94] (9)	Petrolatum-soaked gauze, placebo	Several trials reported benefits with different topical growth factors. However, the incremental benefit of these biological agents over less expensive standard wound care remains uncertain.	Positive
Saline spray containing aloe vera, silver chloride and decyl glucoside, saline, whirlpool	Moore, 2008[92] (3)	No wound cleansing, or different wound cleansing solutions, or different cleansing techniques (Isotonic saline solution, tap water, no whirlpool)	No statistically significant change in healing was seen when water was compared with saline. No statistically significant change in healing was seen for ulcers cleansed with, or without, a whirlpool.	Neutral
Topical negative pressure	van den Boogaard,	Cadexomer-iodine impregnated dressing, papin-	TNP has not proven to be more effective than	Neutral

	2008[97] (5)	urea ointment, hydrocolloid, alginate, acetic acid, sodium hypochlorite, gauzes wetted in a saline solution or Ringer's solution, nitrofuralum	various control interventions.	
Topical negative pressure (vacuum assisted wound closure)	Pham, 2003[104] (17)	Moist gauze, healthpoint system, opposite dressing, standard pressure, bolster dressing, conventional dressing	For management of pressure sores and ulcers, no difference could be detected between vacuum assisted wound closure and use of traditional gauze dressings or the healthpoint system. Vacuum assisted wound closure therapy appeared to be more effective than Opsite and bolster dressings in skin graft management. Patients managed with vacuum assisted wound closure had increased rate of reepithelialisation and fewer patients required repeat split thickness skin graft to the same site. Vacuum assisted wound closure was more effective at treating various chronic and complex wounds than wet-to-moist gauze, as there was a significantly greater reduction in wound volume, depth and treatment duration.	Neutral
Pressure ulcers - proportion of patients with healed wounds (2 reviews)				
Collagenase, dressings	Reddy, 2008[94] (1)	Hydrocolloid dressing	No other dressing was superior to alternatives. Little evidence supports the use of a specific support surface or dressing over other alternatives.	Neutral
Electric current, electromagnetic therapy	Reddy, 2008[94] (3)	Placebo, Standard care	There is little evidence to support adjunctive therapies compared with standard care.	Neutral
Hydrogel, hydrocolloid, hydrocolloid alginate, polyurethane foam	Reddy, 2008[94] (12)	Transparent absorbent acrylic dressing, hydrogel dressing, copolymer	No other dressing was superior to alternatives. Little	Neutral

dressings		membrane, moist povidone-iodine gauze, moist saline gauze, collagen, change indicator, polyhema	evidence supports the use of a specific support surface or dressing over other alternatives.	
Laser therapy + moist saline gauze	Reddy, 2008[94] (1)	Moist saline gauze	There is little evidence to support adjunctive therapies compared with standard care.	Neutral
Monochromatic phototherapy, UV light	Reddy, 2008[94] (2)	Placebo, placebo UV light	There is little evidence to support adjunctive therapies compared with standard care.	Neutral
Oxyquinoline, radiant heat, soft silicone, hydrogel or foam, active ointment with live yeast derivative, topical insulin (dressings)	Reddy, 2008[94] (6)	Lanolin, petrolateum, hydrocolloid or alginate dressing, hydropolymer dressing, transparent film, standard care, placebo	No other dressing was superior to alternatives. Little evidence supports the use of a specific support surface or dressing over other alternatives.	Neutral
Resin salve absorbent dressings	Reddy, 2008[94] (1)	Sodium caroxymethylcellulose hydrocolloid polymer dressing	No other dressing was superior to alternatives. Little evidence supports the use of a specific support surface or dressing over other alternatives.	Neutral
Specialized foam mattress, alternating pressure mattress	Reddy, 2008[94] (3)	Water mattress, alternating pressure overlay, alternating pressure mattress	No clear evidence favored one support surface over another.	Neutral
Ultrasound	Reddy, 2011[78] (2)	Sham ultrasound	Unclear whether therapeutic ultrasound is more effective than sham ultrasound at increasing the number of sores healed.	Indeterminate
<u>Mixed chronic wounds – ulcer healing (5 reviews)</u>				
Adhesive zinc oxide tape	Bradley, 1999a[111] (1)	Hydrocolloid dressing	Study positive for adhesive zinc oxide tape vs traditional treatment	Positive*
Dextranomer polysaccharide beads or paste, cadexomer iodine polysaccharide beads or paste	Bradley, 1999a[111] (18)	Traditional/control treatment, other debriding agents	3/9 positive for dextranomer vs control. 2/9 positive for control vs dextranomer. 3/9 positive for cadexomer	Neutral*
	Bradley, 1999a[111] (4)	Other debriding agents: collagenase and streptokinase/streptodornase, hydrogel	1/3 studies positive for dextranomer polysaccharide vs hydrogel	Neutral*
	Bradley, 1999a[111]	Dextranomer polysaccharide, Hydrogel	No studies showed statistically significant	Neutral*

	(2)		effect between cadexomer vs comparators	
Enzymatic agents (topical)	Bradley, 1999a[111] (5)	Traditional or control treatment	No significant differences in studies	Neutral*
	Bradley, 1999a[111] (1)	Enzymatic agents	No significant differences in studies	Neutral*
Hydrogels dressings	Bradley, 1999a[111] (4)	Control or traditional treatment	1/4 studies positive for hydrogels vs control or traditional treatment	Neutral*
	Bradley, 1999a[111] (1)	Hydrogels	No statistically significant difference between the two treatments	Neutral*
Hyperbaric oxygen therapy	Wang, 2003[105] (57)	Standard wound care	Hyperbaric oxygen therapy may be beneficial as an adjunctive therapy for chronic non healing diabetic wounds.	Positive
No-sting barrier film bandages	Schuren, 2005[100] (9)	Conventional treatment, zinc oxide, petrolatum, hydrocolloid, placebo, no film	Compared with no treatment or a placebo, liquid film-forming acrylate has a significant impact on the integrity of the peri-wound skin.	Positive
Silver releasing dressing, non-releasing silver-activated charcoal dressing, hydrocolloid-silver-Vaseline impregnated dressing, silver coated dressing, hydrocolloid-silver releasing dressing, silver releasing-foam dressing	Lo, 2008[91] (14)	Non-silver dressings including placebo (hydropolymer foam dressing, calcium alginate dressing, foam dressing)	Silver-releasing dressings show positive effects on infected chronic wounds.	Positive*
Topical Negative Pressure (open-cell foam dressing with continuous suction)	Evans, 2001[107] (2)	Normal wet-to-moist gauze dressings (with an occlusive dressing used to secure the gauze) changed 3 times daily.	Two small trials provide weak evidence to suggest that topical negative pressure may be superior to saline-gauze dressings in terms of wound healing. It was not possible to determine the optimum topical negative pressure regimen.	Positive*
Infected surgical wounds – wound area/size reduction (2 reviews)				
Alginate dressings	Lewis, 2001[108] (2)	Traditional gauze dressing	There was no significant difference between any of the	Neutral*

			groups with regard to the reduction in wound area and volume.	
Foam dressings	Vermeulen, 2012[71] (CR: Vermeulen, 2005[113], Vermeulen, 2004[114]) (1)	Gauze and antiseptic agents	The difference in reduction in wound size at 4 weeks was not significantly different. There is no clear evidence of a difference between gauze and foam in terms of healing.	Neutral*
<u>Infected surgical wounds – time to healing or rate of healing (5 reviews)</u>				
Alginate dressings	Vermeulen, 2012[71] (CR: Vermeulen, 2005[113], Vermeulen, 2004[114]) (2)	Gauze and sodium hypochlorite, gauze and povidone iodine	Only 2 small trials have compared alginate with gauze for surgical wounds healing rate.	Indeterminate*
Aloe vera dermal gel (topical)	Dat, 2012[68] (1)	Standard care	Inconclusive. The poor quality of the included trials indicates that the trial results must be viewed with extreme caution as they have a high risk of bias.	Indeterminate*
Dextranomer polysaccharide beads dressings	Lewis, 2001[108] (1)	Traditional gauze dressings + Eusol	No conclusions could be drawn from the results.	Indeterminate*
	Lewis, 2001[108] (1)	Silicone dressings	There was no significant difference in the mean healing time for wounds treated with either dextranomer polysaccharide beads or silicone foam dressings.	Neutral*
Foam dressings	Vermeulen, 2012[71] (CR: Vermeulen, 2005[113], Vermeulen, 2004[114]) (5)	Gauze and antiseptic agents, moistened gauze	None of the trials showed a statistically significant difference in time to healing between foam and gauze. All of these trials were small and we could not be confident that any of them had blinded assessment of outcomes. There is no clear evidence of a difference between gauze and foam in	Neutral*

			terms of healing.	
	Vermeulen, 2012[71] (CR: Vermeulen, 2005[113], Vermeulen, 2004[114]) (1)	Dextranomer beads	There was no significant difference in time to complete healing. There is only 1 poor quality trial.	Neutral*
Gauze + aloe vera dressings	Vermeulen, 2012[71] (CR: Vermeulen, 2005[113], Vermeulen, 2004[114]) (1)	Gauze	Mean time to healing was reported as significantly greater with Aloe Vera compared with gauze, however the differential loss to follow up means that these results are not interpretable.	Indeterminate*
Honey (topical)	Moore, 2001[109] (1)	Antiseptics and systemic antibiotics	Study positive for honey vs other interventions. Confidence in a conclusion that honey is a useful treatment for superficial wounds or burns is low	Positive
Hydrocolloid dressings	Lewis, 2001[108] (1)	Traditional gauze dressings with povidone iodine	There was no significant difference in median healing time between the hydrocolloid groups combined and the gauze treatment group	Neutral*
	Vermeulen, 2012[71] (CR: Vermeulen, 2005[113], Vermeulen, 2004[114]) (1)	Gauze	There was no difference in median time to healing between the groups.	Neutral*
Plaster casting	Vermeulen, 2012[71] (CR: Vermeulen, 2005[113], Vermeulen, 2004[114]) (1)	Elastic compression bandage	Lower limb amputation wounds healed significantly more quickly	Positive*
Polyurethane foam and sheets dressings	Vermeulen, 2012[71] (CR: Vermeulen, 2005[113],	Alginate and polyurethane sheets dressing	There is insufficient evidence to inform on the relative effects of foam and alginate dressing on healing.	Indeterminate*

	Vermeulen, 2004[114]) (1)			
Silicone elastomer foam dressings and polyurethane foam dressings	Lewis, 2001[108] (5)	Traditional moist gauze	No significant differences between interventions.	Neutral*
Topical negative pressure	Ubbink, 2008b[96] (13)	Hydrocolloids (gel, dressings), hydrogels, alginates (dressing), acetic acid, eusol, sodium hypochlorite gauze dressing, saline gauze, accuzyme, iodosorb, compression dressing, bolster dressing, gauze in ringers solution	In chronic and diabetic wounds, topical negative pressure did not allow earlier complete wound healing. It was, however, associated with a 1–10 day reduction in the time needed to prepare the wound for secondary closure surgery.	Neutral*
<u>Infected surgical wounds – ulcer healing (2 reviews)</u>				
Dextranomer polysaccharide beads dressings	Lewis, 2001[108] (1)	Traditional gauze dressings and Eusol	No conclusions could be drawn from the results.	Indeterminate*
	Vermeulen, 2012[71] (CR: Vermeulen, 2005[113], Vermeulen, 2004[114]) (1)	Gauze and Eusol	There was no significant difference between dextranomer and Eusol gauze in terms of number of wounds healing. There is insufficient evidence from one trial on the comparative effects of dextranomer bead dressing and Eusol soaked gauze	Indeterminate*
Polyurethane foam dressings	Lewis, 2001[108] (2)	Alginate dressings, silicone dressings,	No conclusions could be drawn.	Indeterminate*
Note: IM = intramuscular, IV = intravenous, SC = subcutaneous, NR = not reported, RCT = randomized clinical trial, UV = ultraviolet.<br>*These are high quality systematic reviews (AMSTAR score ≥8).				
